# Enhancing cereal productivity via nitrogen use efficiency: from conventional breeding to modern genomics

**DOI:** 10.3389/fgene.2026.1822936

**Published:** 2026-06-22

**Authors:** Anand Kumar, Pandiyan Muthuramalingam, Laxmidas Verma, Padmini Ganga, Reetesh Kumar, Manikandan Ramesh, Hyunsuk Shin

**Affiliations:** 1 Faculty of Agricultural Sciences, GLA University, Mathura, Uttar Pradesh, India; 2 Department of GreenBio Science, College of Agriculture and Life Sciences, Gyeongsang National University, Jinju, Republic of Korea; 3 Department of Genetics and Plant Breeding, Acharya Narendra Deva University of Agriculture and Technology, Ayodhya, India; 4 Department of Biotechnology and Bioengineering, School of Biosciences & Technology, Galgotias University, Greater Noida, India; 5 Department of Biotechnology, Alagappa University, Karaikudi, Tamil Nadu, India

**Keywords:** conventional breeding, CRISPR/Cas9, genomics, MAS, miRNA, NUE, QTLs, TFs

## Abstract

Cereal crops constitute the primary staple foods in India and worldwide, forming the foundation of food security by supplying essential nutrients, carbohydrates, proteins and minerals that are essential for human health. Cereal productivity is largely determined by the availability and efficient utilization of nutrients, particularly nitrogen (N), which is a vital macronutrient involved in chlorophyll biosynthesis, amino acid formation and overall plant growth and yield. However, nitrogen remains the most limiting nutrient in agricultural crops due to its highly dynamic nature and susceptibility to losses through leaching, volatilization, and denitrification. To address these constraints, researchers have adopted integrated strategies encompassing genetic, physiological and agronomic approaches. Conventional breeding programs focus on the selection and hybridization of genotypes with superior nitrogen use efficiency (NUE), targeting key traits such as improved root architecture including root length, density and branching, efficient root transporters for nitrate and ammonium uptake and enhanced nitrate assimilation mechanisms. In addition, canopy architecture and photosynthetic efficiency play a crucial role in optimizing nitrogen utilization, ultimately contributing to higher grain yield and improved grain protein content. Molecular breeding, including quantitative trait loci (QTL) mapping has emerged as a powerful approach for unraveling the complex genetic architecture of NUE related traits. The identification of major QTLs governing root system architecture, nitrogen uptake, assimilation enzymes and yield components facilitates marker assisted selection (MAS) for the rapid introgression of favorable alleles into elite cultivars. Furthermore, integrating biological nitrogen inhibitors (BNIs) as a sustainable agronomic strategy helps reduce nitrogen losses by suppressing nitrification in soil. Recent advances in genomic technologies, including the study of transcription factors, microRNAs (miRNAs), and clustered regularly interspaced short palindromic repeats (CRISPR/Cas9) based genome editing, have revolutionized cereal crop improvement. These tools enable precise regulation and modification of genes involved in nitrogen metabolism, such as nitrate transporters and glutamine synthetase (GS), thereby enhancing nitrogen assimilation pathways. Transcription factors, in particular, play a pivotal role in regulating gene expression networks associated with nitrogen uptake, transport and utilization. Modern approaches significantly enhance the potential for developing high-yielding cereal varieties with improved NUE, reduced fertilizer dependency, and better environmental sustainability.

## 1 Introduction

Cereals have historically been a crucial element of human existence, acting as the principal food source for billions of people. Cereals belong to the Poaceae family and are primarily cultivated for their grains, which are rich in carbohydrates, proteins, dietary fiber, vitamins and minerals (Ahmad et al., 2024). The most frequently consumed grains encompass wheat, rice, maize, barley, oats, rye, and millets, which encounter several obstacles that jeopardize their production and sustainability (McKevith, 2004; Ibrahim et al., 2024; Mehta et al., 2024). Among these, the widespread practice of continuous use of monocropping has caused soil degradation, leading to nutrient depletion and erosion, which subsequently diminishes the production of crops (Mihrete and Mihretu, 2025; Patel et al., 2025). Researchers and agricultural experts are devising creative techniques to enhance cereal output to tackle these difficulties (Rastogi et al., 2024).

So, these efforts emphasizing research, adopting innovative farming techniques and enacting sustainable regulations are crucial to ensuring a stable and secure food supply for future generations (Pretty, 2008). Cereals are the cornerstone of world agriculture, and their ongoing enhancement and adaptation to evolving environmental conditions will be essential for satisfying the demands of an expanding population (Saleem et al., 2024; Carr et al., 2024). Furthermore, cereal crops are increasingly confronted with the elevated demand and reliance on nitrogen fertilizers. In this context, sustainable intensification techniques must incorporate both NUE, as high nitrogen inputs can lead to fertilizer leaching, groundwater contamination and diminished system sustainability (Quemada and Gabriel, 2016). Future breeding and management strategies should concentrate on managing the nitrogen-water nexus to reduce environmental trade-offs while ensuring crop output. Furthermore, NUE in cereal crops is essential for maintaining agricultural productivity while reducing environmental effects. As nitrogen is a vital nutrient for plant growth and development, it plays a significant role in photosynthesis, protein synthesis and biomass production, thereby directly influencing NUE (Ibn et al., 2024). Excessive application of nitrogen fertilizers leads to environmental contamination, greenhouse gas emissions and soil damage. Consequently, enhancing NUE in cereals is crucial for augmenting crop yields while reducing nitrogen losses via leaching, volatilization and denitrification (Andualem et al., 2024). Principal cereals, including wheat, rice, maize, barley and millets, are substantial consumers of nitrogen fertilizers. Rice and maize demonstrate notably elevated nitrogen requirements owing to their widespread cultivation and high yield (Ladha et al., 2005; Vinci et al., 2022; Ibrahim et al., 2024). The efficiency of nitrogen in various crops vary under different conditions and are influenced by factors such as genetic traits, soil quality, climate and agricultural practices. NUE is typically defined as the grain yield per unit of nitrogen supplied, encompassing uptake, assimilation and utilization efficiency (Hirel et al., 2011). Optimizing NUE requires a holistic strategy encompassing meticulous nitrogen management, enhanced soil health, cultivation of nitrogen efficient crop varieties and the implementation of precision agriculture technologies (Sandhu et al., 2021; Govindasamy et al., 2023).

In this context, traditional breeding is a crucial method for enhancing NUE. This procedure entails selecting and hybridizing high-NUE genotypes to create crop varieties that more effectively absorb and utilize nitrogen. Phenotypic selection, relying solely on traditional breeding methods is frequently used to identify plants with desirable traits (Javed et al., 2022). Conventional breeding is constrained by protracted breeding cycles and vulnerability to environmental fluctuations. These challenges highlight the need for progress in molecular breeding and biotechnology, which have empowered researchers to identify and modify genes involved in nitrogen uptake and assimilation (Yali and Mitiku, 2024). Furthermore, MAS has transformed crop breeding by facilitating the accurate identification of genetic markers linked to NUE traits. Techniques such as QTL mapping, genome-wide association studies (GWAS), genomic selection, transcription factors (TFs), miRNAs and CRISPR have been pivotal in identifying genetic regions associated with nitrogen uptake, transport and assimilation (Elrys et al., 2022; Mallikarjuna et al., 2022).

Building on these advances, genetic engineering has emerged as a pivotal tool for directly modifying genes linked to NUE. Augmenting the expression of nitrogen transporters, including nitrate transporters (NRT1, NRT2) and ammonium transporters (AMT), has been shown to enhance nitrogen absorption efficiency (Agrama, 2006). Modulation of genes regulating nitrogen assimilation, such as GS and nitrate reductase (NR), has been shown to improve nitrogen utilization efficiency (NUtE) (Lebedev et al., 2021; Biradar et al., 2024). Moreover, TFs are pivotal in regulating NUE by influencing the expression of genes involved in nitrogen uptake, assimilation, transport and remobilization. NUE is a quantitative feature that influences the efficacy with which plants utilize available nitrogen for growth, biomass accumulation and grain production, representing a complicated trait (Zhao et al., 2023; Kumar et al., 2024). Engineering TFs such as NIN-like proteins (NLP) and DNA-binding with one finger (Dof) has further optimized nitrogen metabolism pathways (Sandhu et al., 2021; Lal et al., 2024).

**FIGURE 1:**
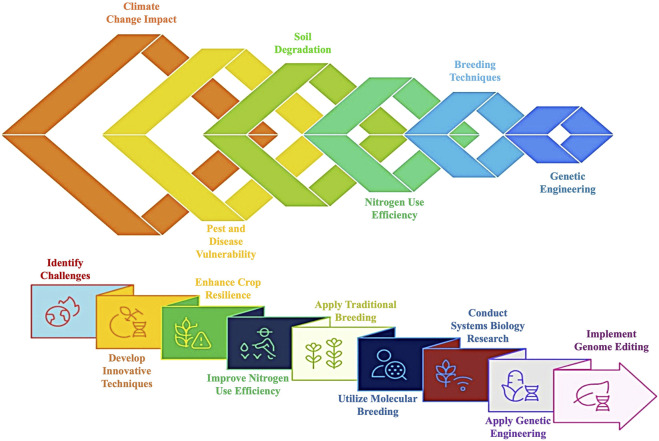
Framework illustrating agricultural challenges and integrated breeding, biotechnology and genome editing strategies for developing climate-resilient and high-yielding varieties.

The advent of genome editing technologies, particularly CRISPR/Cas9 has opened remarkable prospects for NUE with unparalleled accuracy and efficacy. CRISPR/Cas9 facilitates the accurate alteration of genes associated with nitrogen intake, assimilation and remobilization. For example, inhibiting negative regulators of nitrogen metabolism can raise NUE, while activating positive regulators can further optimize nitrogen usage. Currently, nitrogen efficient crops can be developed by precisely adjusting gene expression *via* genome editing which is tailored to specific environmental circumstances (Fiaz et al., 2021; Liu et al., 2022). The success of genome editing in enhancing NUE underscores its potential as a revolutionary technique for promoting sustainable agriculture (Fiaz et al., 2021). The details are illustrated in Figure 1, which depicts the interrelated challenges and strategic approaches in contemporary crop improvement, highlighting the dynamic interplay between environmental stressors and advanced breeding technologies. Critical issues, including climate change, soil degradation, pest and disease susceptibility and nitrogen use inefficiency, are presented as interconnected challenges that affect agricultural productivity and sustainability (Dave et al., 2024; Mihrete and Mihretu, 2025; Patel et al., 2025). A series of remedies is outlined, beginning with identifying issues and developing creative methodologies, followed by enhancing crop resilience and improving NUE. The image underscores the amalgamation of traditional breeding, molecular breeding, systems biology research and sophisticated genetic engineering methods, including genome editing, as vital elements of a holistic crop enhancement strategy. This visual framework emphasizes the need for a multidisciplinary approach to ensure food security and agricultural sustainability amid global climate and environmental challenges.

## 2 Traits affecting NUE

NUE is mainly governed by nitrogen uptake efficiency (NUpE), NUtE and nitrogen recovery efficiency (NRE) (Kunwar et al., 2025; Reddy et al., 2025). Among these, NUpE reflects the plant’s capacity to acquire nitrogen from the soil and is largely influenced by root traits such as root length, architecture and transporter activity. It is also affected by soil nitrogen availability, external fertilization, plant adaptive plasticity and plant-microbe interactions that regulate nitrogen transformation and diffusion in the rhizosphere (Bhardwaj et al., 2021; Zhang B. et al., 2022; Kumar et al., 2022; Muller et al., 2023). In contrast, NUtE represents the efficiency with which absorbed nitrogen is converted into biomass and grain yield and is governed by photosynthetic capacity, enzymatic activity and carbon-nitrogen metabolic coordination (Jan et al., 2025; Liu H. et al., 2025). On the other hand, NRE integrates both uptake and utilization processes and indicates the proportion of applied nitrogen that is effectively recovered by the plant, thereby linking nitrogen dynamics with crop productivity (Zou et al., 2024; Ali et al., 2025). Together, these components determine overall NUE, which is often more strongly driven by NUpE, as it defines the amount of nitrogen available for subsequent utilization, while NUtE determines how efficiently that nitrogen is converted into economic yield (Ibn et al., 2025). This is supported by experimental evidence where significant genotypic differences in NUE and NUpE were observed, primarily due to inherent genetic variation in root growth and nitrogen acquisition efficiency (Vazquez-Carrasquer et al., 2021). For instance, genotypes such as XJ19-1, KN9204, WR9603 and XJ138-1 developed stronger root systems and higher root biomass, particularly in the topsoil layer, enabling enhanced nitrogen uptake and improved NUE compared to S4185 (Wang et al., 2023). Notably, KN9204 exhibited significantly higher root dry weight under both high and low nitrogen conditions, resulting in superior NUpE and productivity (Wang et al., 2011). These genotypes also demonstrated efficient nitrogen assimilation and remobilization, contributing to higher grain yield and biomass across environments (Padhan et al., 2023).

Similarly, nitrogen uptake dynamics are strongly influenced by nitrogen availability. Both high and low NUE cultivars exhibit maximum NUpE under low nitrogen supply (0.2 mM), which declines with increasing nitrogen levels due to the downregulation of high-affinity transport systems (Mahboob et al., 2023). At moderate nitrogen levels (2 mM), nitrate (NO_3_^−^) uptake predominates in the root mature zone, whereas ammonium (NH_4_^+^) uptake occurs mainly in the meristematic and elongation zones. However, high-NUE cultivars exhibit significantly greater NH_4_^+^ uptake in the root tip and enhanced NO_3_^−^ uptake in the mature zone, indicating better spatial coordination and higher transporter activity, which ultimately improves overall nitrogen acquisition (Li et al., 2024). In addition to uptake, NUtE plays a crucial role in maximizing crop yield under varying nitrogen conditions. Studies in maize have shown that reduced nitrogen supply decreases total dry matter and nitrogen uptake, but enhances NUpE, NUtE and overall NUE due to improved nitrogen remobilization and efficient utilization under limited nitrogen availability (Zhu et al., 2025; Ma et al., 2025). The significant genotype × nitrogen interaction further highlights the genetic variability in nitrogen acquisition and utilization traits. NUE was found to be positively correlated with grain yield, biomass, total nitrogen uptake and NUpE, confirming that uptake efficiency is a major determinant of NUE, while the relative contribution of NUtE increases under high nitrogen conditions (Kunwar et al., 2025; Huang et al., 2025). Moreover, agronomic interventions such as controlled-release nitrogen fertilizers (CRNF) enhance soil nitrogen availability throughout crop growth, reduce leaching losses, and improve plant nitrogen accumulation, thereby strengthening yield formation (Ali et al., 2025).

Furthermore, NRE reflects the plant’s ability to utilize applied nitrogen effectively and is closely associated with yield response. NRE typically shows a quadratic response to nitrogen application, where efficiency increases up to an optimal level and declines beyond it due to saturation of uptake capacity and increased nitrogen losses through leaching or volatilization (Govindasamy et al., 2023; Zou et al., 2024). Soil fertility status also modulates this response, as nutrient balance influences nitrogen uptake and utilization efficiency. Optimal nitrogen application rates therefore vary with soil fertility levels to achieve a balance between maximum yield and environmental sustainability. Additionally, integrated management practices combining appropriate irrigation and nitrogen application enhances photosynthesis, NR activity and dry matter accumulation, ultimately leading to increased grain yield and improved NUE (Zhu et al., 2022).

### 2.1 Root architecture

Characteristics associated with nitrogen uptake efficiency in cereal crops are essential for improving NUE and fostering sustainable agricultural practices, as root architecture significantly influences a plant’s capacity to assimilate nitrogen from the soil. Understanding the relationship between root traits and nitrogen uptake efficiency can facilitate the development of crop varieties that use nitrogen more efficiently (Govindasamy et al., 2023; Wang et al., 2023). An advanced root system facilitates enhanced access to nitrogen, especially in soils with limited nitrogen supply. Root architecture denotes the spatial configuration of a plant’s root system, including attributes such as root length density, branching patterns, root hair formation, lateral root development, root angle, root depth and the capacity to establish symbiotic relationships with beneficial microbes (Freschet et al., 2021; Kalra et al., 2024). Among these, root length density (RLD) is a significant quantitative feature influencing nitrogen absorption. An enhanced root length density allows plants to explore a larger soil volume, thereby increasing the likelihood of accessing nitrogen sources (Garnett et al., 2009). Cereal crops like wheat, rice and maize with elevated root length density are more proficient at obtaining soil nitrogen, particularly under nitrogen deficient circumstances. Emphasizing elevated root length density in breeding initiatives can augment nitrogen absorption and markedly enhance NUE. The formation of root hairs is crucial for nutrient absorption, especially nitrogen (Sun et al., 2021). Root hairs augment the root surface area, enhancing interaction with soil particles and promoting the uptake of nitrate and ammonium ions. In cereal crops, differences in root hair density and length can markedly affect nitrogen uptake efficiency (Zhang H. et al., 2023). Research indicates that augmenting root hair formation through biotechnological methods and genetic selection may enhance NUE in crops such as wheat and maize (Saengwilai et al., 2021; Wang et al., 2023).

Additionally, researchers have found several genes and hormones, including auxins and cytokinins, which govern lateral root formation in cereals. Focusing on these routes in breeding programs offers an opportunity to enhance root architecture and improve nitrogen uptake efficiency. Moreover, root depth and growth angle are essential for efficient nitrogen acquisition (Maqbool et al., 2022; Alrajhi et al., 2024). Cereal crops having deeper root systems can access nitrogen that has leached into the lower soil strata, whereas those with shallower roots are more efficient at absorbing surface applied fertilizers (Yadav et al., 2023). A pronounced inclination toward deeper rooting is especially beneficial in situations where nitrogen is located at greater soil depths. Acquiring a profound understanding of the genetic determinants that influence root depth and angle could yield effective techniques for cultivating nitrogen efficient cereal varieties suited to diverse soil conditions (Whetton et al., 2022).

Additionally, symbiotic interactions between plant roots and beneficial microorganisms, such as mycorrhizal fungi and nitrogen fixing bacteria are essential for improving nitrogen uptake efficiency (Xiong et al., 2021). Mycorrhizal fungi enhance nutrient absorption, especially nitrogen, by extending their hyphae into the soil and facilitating nutrient transfer to the host plant. Enhancing mycorrhizal associations in cereal crops through breeding or biofertilizer application can markedly increase nitrogen uptake (Khan et al., 2022). Nitrogen-fixing bacteria, like Azospirillum and Rhizobium, enhance nitrogen availability in cereal crops. Furthermore, environmental variables such as soil composition, water accessibility, and microbial activity interact with root characteristics to affect nitrogen uptake efficiency (Aasfar et al., 2021; Katiyar et al., 2022). In sandy soils, cereals possessing broader and deeper root systems are better able to acquire nitrogen, but compacted soils may impede root penetration. Moreover, dry conditions might inhibit root development, hence diminishing nitrogen absorption. Understanding the interplay between root characteristics and environmental conditions are crucial for optimizing nitrogen use efficiency across diverse agricultural systems (Li et al., 2022; Bodner et al., 2021). Future research should concentrate on integrating targeted root trait enhancement with holistic nitrogen management strategies to optimize productivity and sustainability (Ciampitti and Lemaire, 2022).

In a study, Bam, Tabbasi, Qods and Mahooti exhibited the highest seminal axile root length under control conditions, whereas Falat and Roshan showed lower values; however, under −7 to −8 stress, root length declined in all cultivars (22%–41%), with greater reductions in sensitive than tolerant types, where Roshan retained the highest and Falat the lowest relative values. Furthermore, total root length decreased, with smaller reductions in tolerant cultivars (8.2%) than in sensitive cultivars (17.9%), indicating that deeper root growth is a key trait for drought tolerance (Rahnama et al., 2024). This occurred mainly due to differences in drought tolerance mechanisms among the cultivars. Under water-deficit stress (−7 to −8), reduced soil moisture limits cell expansion, root elongation and overall plant growth, leading to a decline in root length (Rahnama et al., 2024). Sensitive cultivars experience greater reductions because they have weaker physiological and morphological adaptations, such as poor osmotic adjustment, lower water retention and less efficient root systems (Zahedi et al., 2025). In contrast, tolerant cultivars like Roshan maintains relatively higher root length due to better stress adaptation such as efficient water uptake, sustained cell turgor and deeper or more robust root architecture (Ranjan et al., 2022). This allows them to explore deeper soil layers for moisture, resulting in smaller reductions in total root length. Hence, deeper and well developed root systems play a crucial role in improving drought tolerance (Wasaya et al., 2018).

### 2.2 Root transporter

Root nitrogen (N) transporter systems are essential for effective nitrogen absorption and overall development in cereal crops (Ohyama, 2010). Nitrogen primarily exists in many forms in both natural and agricultural ecosystems, chiefly as nitrate (NO_3_^−^) and ammonium (NH_4_^+^) which are absorbed by plant roots via specialized transporter proteins (Muratore et al*.,* 2021). A comprehensive understanding of root nitrogen transporter systems is essential for enhancing NUE in cereals, hence mitigating overuse and limiting environmental pollution (Kumari et al., 2022). Plants facilitate nitrogen absorption mainly via two transporter systems as nitrate transporters and ammonium transporters. Nitrate transporters are categorized into two prominent families as NRT1 (Nitrate Transporter 1/Peptide Transporter Family-NPF) and NRT2 (High-Affinity Nitrate Transporters), while ammonium transporters are part of the AMT (Ammonium Transporter) family. These transport mechanisms function under varying nitrogen concentrations and are governed by internal nitrogen requirements, external nitrogen supply and diverse environmental variables (Buchner and Hawkesford, 2014; Nedelyaeva et al., 2024). The NRT1 family mostly operates under low affinity circumstances, indicating heightened activity when soil nitrate levels are elevated. Certain members of the NRT1 family, including *NRT1.1* (also known as *CHL1*), function as dual-affinity transporters that are able to alternate between high and low-affinity transport modes based on nitrate availability. This adaptability enables plants to respond effectively to varying soil nitrogen concentrations (Fang et al., 2021; Jia et al., 2023; Islam et al., 2022). Conversely, the NRT2 family functions in high affinity conditions, focusing on nitrate absorption when nitrogen is scarce. The NRT2 transporters typically operate in tandem with NAR2 proteins which are crucial for their proper functionality (Muratore et al., 2021). Furthermore, peptide transporters (PTRs) enable the uptake of organic nitrogen molecules, including short peptides and amino acids. Furthermore, aquaporins, conventionally recognized for their function in water transport, have also been demonstrated to facilitate the translocation of ammonium and nitrate across root cell membranes. Additionally, proton pumps and ion channels facilitate nitrogen absorption by sustaining the electrochemical gradients essential for the transfer of nitrate and ammonium (Muratore et al., 2021; Liu et al., 2024). The control of root nitrogen transporters is exceedingly intricate, involving numerous signaling pathways. Nitrate and ammonium signaling is regulated by TFs, kinases, and metabolic feedback mechanisms that synchronize nitrogen absorption with the plant’s growth requirements.

Moreover, the *NRT1.1* transporter functions as both a nitrate sensor and a nitrate transporter, affecting root architecture and shoot growth in relation to nitrogen availability (Wang P. et al., 2021; Hu et al., 2021). The activity of ammonium transporters is regulated by phosphorylation, which modifies their function in response to the plant’s internal nitrogen levels. Environmental variables, including soil pH, temperature and microbial interactions, strongly influence the expression and efficacy of nitrogen transporters. Acidic soils enhance ammonium availability and diminish nitrate uptake, whereas alkaline conditions promote nitrate absorption over ammonium (Dubey et al., 2021; Shilpha et al., 2023).

Additionally, genetic and biotechnological methods have been used to improve nitrogen uptake efficiency in wheat by targeting root nitrogen transporters. The overexpression of high-affinity nitrate transporters, like NRT2.1, has been shown to enhance nitrate uptake and assimilation in rice and wheat. Likewise, *AMT* genes have increased ammonium absorption and enhanced NUE in maize and barley (Tiong et al., 2021; Esmaeilzadeh et al., 2023). Understanding the link between nitrogen transporters and plant-microbe interactions will be essential for developing nitrogen-efficient cropping systems that enhance global food security.

### 2.3 Nitrate assimilation

Nitrate absorption and assimilation are essential processes in cereal crops. Nitrogen is initially absorbed from the soil by the roots via specialized nitrate transporters (Mahboob et al., 2023). Following absorption, nitrate is processed in root cells and sent through the xylem to the stem and other plant regions for storage. The dynamic interplay between NRT1 and NRT2 transporters at the root interface allows plants to adjust nitrate absorption in response to environmental variations and nitrogen supply (Nacry et al., 2013; Htwe and Ruangrak, 2021; Akhtar et al., 2024). Subsequent to absorption and initial digestion, diverse organic nitrogen components such as proteins, amino acids and nucleotides are produced, markedly augmenting crop productivity. The initial phase of nitrate assimilation entails the reduction of nitrate to nitrite, facilitated by the enzyme NR (Zayed et al., 2023). NR serves as a crucial regulatory juncture in nitrate metabolism, with its activity meticulously regulated by several elements including light, carbon availability and the plant’s internal nitrogen status. NR regulation occurs at both transcriptional and post-translational levels to enhance nitrate assimilation in accordance with the plant’s physiological requirements (Htwe and Ruangrak, 2021).

Nitrite, a highly reactive and possibly deadly compound, is swiftly carried to the chloroplasts in leaves or the plastids in roots, where it is transformed into ammonium (NH_4_^+^) by the enzyme nitrite reductase (NiR). This procedure is essential to prevent the buildup of excess nitrite, which could jeopardize cellular integrity. The activity of nitrite reductase is meticulously regulated to sustain an appropriate equilibrium in the nitrogen assimilation pathway (Mohn, 2019; Ravazzolo, 2019). Furthermore, AMTs play a crucial role in plant nitrogen acquisition by mediating the uptake of ammonium (NH_4_^+^) from the soil under both low and high-affinity conditions, thereby ensuring efficient absorption across diverse environmental situations. Once inside the plant, ammonium is rapidly assimilated into organic forms primarily through the glutamine synthetase/glutamate synthase (GS/GOGAT) pathway. In this process, glutamine synthetase catalyzes the incorporation of ammonium into glutamate to form glutamine, while glutamate synthase transfers the amide group from glutamine to 2-oxoglutarate, producing two molecules of glutamate (Zhang J. et al., 2021; Kumar et al., 2024). This cycle is fundamental for amino acid biosynthesis and overall nitrogen metabolism. Additionally, alternative pathways, such as asparagine synthetase (AS), contribute to ammonium assimilation and nitrogen storage. While AMTs are essential for nitrogen acquisition, excessive ammonium uptake can lead to toxicity, metabolic imbalances, and root acidification. Moreover, beyond primary nitrate and ammonium transport systems, several secondary transporters also influence root nitrogen uptake efficiency (Liao et al., 2022; Fortunato et al., 2023). This mechanism transforms glutamine and aspartate into asparagine, a crucial nitrogen storage compound in plants. Asparagine serves as a crucial nitrogen transport form that can be rapidly mobilized throughout various growth stages, such as seed development and grain filling in cereals (Kaur et al., 2021). The assimilation of nitrate by living organisms is an energy-demanding process that necessitates reducing equivalents like NADH or NADPH. The availability of these molecules is predominantly contingent upon photosynthesis, underscoring the intricate interrelationship between the nitrogen and carbon cycles in plants (Tu et al., 2023).

The reduction of nitrate is regulated by many signaling molecules, including nitric oxide, cytokinin, phytohormones and abscisic acid, which modulate the expression of essential enzymes in nitrogen metabolism (Singhal et al., 2021). Moreover, environmental conditions significantly influence the efficacy of nitrate assimilation. Microbial activity and cell concentrations in the soil regulates the availability of soil nitrates (sufficient, inadequate, or pH neutral), whereas temperature influences metabolism and nitrate uptake in cereals (Barłóg et al., 2022). Nitrogen production occurs during drought conditions; nevertheless, it is characterized by low nitrogen ß partitioning within the plant, hence limiting the potential for nitrate assimilation. Excess nitrogen can impede nitrate mobility, leading to pollution and eutrophication, and diminish NUE (Dollete et al., 2024). Nitrate assimilation is essential for cereal crops, influencing NUtE, biomass production and grain yield. The sequential conversion of nitrate to ammonium, which is then incorporated into organic molecules, is a regulated, energy-demanding process (The et al., 2021).

### 2.4 Canopy architecture and photosynthesis

Photosynthesis and canopy architecture greatly affect plant growth and yield in field crops, particularly in grain varieties (Stewart et al., 2003). The primary elements of the plant canopy, comprising leaves, stems and reproductive structures, dictate solar interception, gas exchange and ultimately the efficacy of photosynthesis (Murchie and Burgess, 2022). Understanding the canopy architecture and photosynthetic processes is crucial for maximizing production, promoting resource efficiency and improving stress resilience in crops. Canopy architecture denotes the three-dimensional configuration of plant organs within a crop stand. Common characteristics of canopy architecture encompass leaf area index (LAI), leaf angle distribution, plant height, internode length, tillering capability and branching patterns (Prashar et al., 2022; Carbajal-Friedrich and Burgess, 2024). These characteristics significantly influence light penetration through the canopy, thereby affecting photosynthesis at both the leaf and whole plant. An optimal canopy structure enhances light capture and minimizes self-shading, thereby increasing carbon assimilation. Consequently, as photosynthesis converts light energy into chemical energy for plant use and its efficiency is strongly governed by canopy architecture, where the interception, diffusion and utilization of light ultimately determine the overall photosynthetic capacity of the crop (Murchie et al., 2023; Burgess et al., 2023). In a canopy, the higher leaves often receive more light than the lower leaves, which stay partially shaded, limiting their photosynthetic activities. An effectively constructed canopy can ensure uniform illumination across its components, resulting in consistent photosynthetic activity (Murchie et al., 1999).

Furthermore, LAI is the paramount metric that determines canopy architecture and photosynthetic efficacy. LAI is defined as the total leaf surface area per unit of ground area and is a critical determinant of light interception. An optimum LAI increases photosynthetic efficiency and minimizes excessive shadowing. In cereals, an LAI is typically considered optimal for balancing light interception and airflow within the canopy (Gara et al., 2021; Yang et al., 2023). Excessive LAI typically results in increased shadowing, reduced photosynthesis in lower leaves and an elevated risk of fungal infections due to inadequate air circulation (Jat et al., 2021; Kabir et al., 2024). Moreover, leaf angle dispersion significantly influences the regulation of internal light distribution inside the canopy. The vertically inclined erectophile leaves facilitate improved light penetration into lower canopy layers, thereby boosting the photosynthetic efficiency of shaded leaves (Niinemets, 2010). This characteristic is especially advantageous in high-density cultivation, such as contemporary wheat and maize hybrids. Conversely, planophile leaves, which are more horizontally oriented, efficiently capture sunlight at the canopy’s apex but tend to shade the lower leaves excessively, thereby diminishing overall canopy photosynthesis (Jaikumar et al., 2021; Moroyoqui Parra, 2022). Additionally, plant height and internode length substantially affect canopy structure and photosynthetic efficiency. Tall plants with elongated internodes can more efficiently harness light in sparse planting settings; however, they are more susceptible to lodging, particularly in high density planting systems (Xue et al., 2016). To mitigate this issue, semi-dwarf wheat and rice cultivars have been created to enhance lodging resistance while ensuring effective light interception (Li et al., 2021). Tillering and branching features are essential elements of canopy architecture, as they dictate the quantity and spatial configuration of leaves within a crop stand. Numerous cereal crops, particularly specific varieties of rice and wheat, produce multiple tillers, forming a denser canopy. Excessive tillering may result in intense competition for sunlight, water and nutrients, hence diminishing overall photosynthetic efficiency (Dixon et al., 2022; Mohapatra et al., 2025). Consequently, efficient breeding techniques must meticulously balance tillering capacity with canopy architecture to optimize resource utilization for optimal productivity.

Moreover, photosynthesis, stomatal conductance and transpiration are meticulously regulated by the dispersion of light inside the canopy. In well-structured canopies, gas exchange is optimized to equilibrate CO_2_ absorption and water loss *via* stomatal management. Stomatal behavior, significantly influenced by microclimate and environmental variables, is essential for sustaining photosynthetic efficiency across diverse development environments (Driever et al., 2023; Nguyen et al., 2023). Progress in breeding has concentrated on developing canopy structures that improve photosynthetic efficiency. The selection of features such as enhanced leaf angles, optimal LAI, and balanced tillering has become a primary focus. Strategic nitrogen applications can augment these to improve photosynthetic efficiency. So, canopy architecture, closely linked to photosynthesis, significantly influences agricultural productivity and resource use efficiency (Gupta et al., 2023). Breeding management and technology advancements to optimize canopy structure will significantly enhance photosynthetic efficiency, stress resilience, and yield in cereal crops.

### 2.5 Grain protein and grain yield

Grain protein content and grain yield are two essential traits in cereal crops, both of which are affected by nitrogen availability and use. The interaction of nitrogen supply, grain yield and grain protein content directly influences both food production quantity and its nutritional quality and broader applicability (Fradgley et al., 2021; Poutanen et al., 2022). Grain yield is the quantity of grain collected per unit area and is closely linked to the plant’s capacity to efficiently assimilate and utilize nitrogen. Sufficient nitrogen supply facilitates increased shoot formation, expanded leaf area and elevated chlorophyll content, all of which enhance photosynthetic efficiency and biomass accumulation (Gooding and Shewry, 2022; Ludemann et al., 2022). Nitrogen facilitates essential cellular processes, including cell division, elongation and tissue differentiation, which are critical for the proper development and filling of grain heads, ultimately resulting in increased yields. Nevertheless, an excessive supply of nitrogen can be productive. Excessive fertilization of plants can result in excessive vegetative development such as leaves and stems, causing lodging, which diminishes total grain yield and complicates harvesting (Hasan et al., 2024). Consequently, attaining equilibrium in nitrogen management is essential for maximizing both grain yield and grain protein content. Grain protein content is essential for the nutrition and processing of cereals such as wheat, barley and rice. The timing and quantity of nitrogen fertilizer applied substantially affect grain protein content (Safdar et al., 2023). Applying nitrogen later in the growing season, during grain filling, can augment protein content, since nitrogen is translocated from other plant parts to the developing grains. Grain yield and grain protein concentration frequently exhibit an inverse correlation, whereby increased grain yield generally leads to diminished grain protein content. This occurs because high-yielding cultivars allocate a greater proportion of their resources to grain production, which may reduce grain protein concentration (Olaniyi, 2024). Plant breeders seek to mitigate this adverse association by discovering genetic loci linked to both elevated yield and high grain protein content. Advanced methodologies such as marker-assisted selection and genome editing are employed to enhance NUE and attain an optimal equilibrium between grain production and protein quality. Additionally, nitrogen availability affects grain yield and protein content, which are regulated by environmental factors like soil type, climate, and microbial activity. Nitrogen is gradually released from organic materials in the soil for plant absorption (Grzyb et al., 2021; Lyu et al., 2024).

### 2.6 Biological nitrogen inhibitor

**FIGURE 2:**
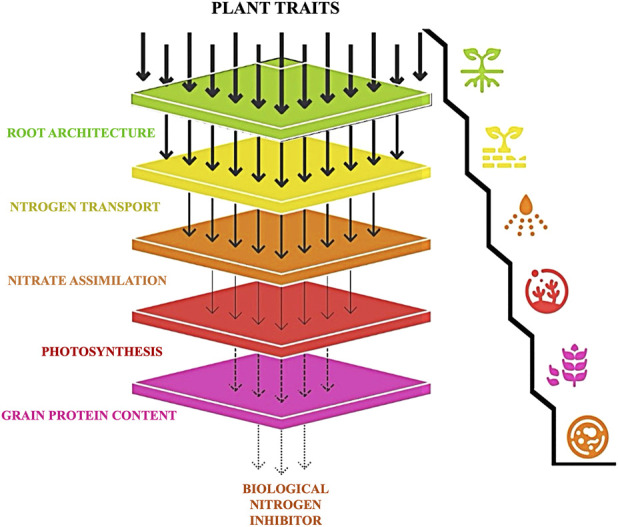
Hierarchical representation of plant traits affecting NUE. The Green Layer represents root architecture controlling nitrogen uptake. The Yellow Layer shows nitrogen transport from roots to shoots. The Orange Layer illustrates nitrate assimilation into usable compounds. The Red Layer highlights photosynthesis regulating carbon fixation, nitrogen metabolism, and biomass production. The Pink Layer indicates grain protein content and nitrogen remobilization. The bottom layer represents biological nitrogen inhibitors that reduce nitrogen loss and improve NUE.

Biological nitrification inhibition (BNI) is a natural phenomenon in which some plant species exude chemicals that suppress the activity of nitrifying bacteria in soil. This inhibition diminishes the conversion of ammonium (NH_4_^+^) to nitrate (NO_3_^−^) (Subbarao et al., 2007). Moreover, BNI is crucial for managing nitrogen cycle which improves NUE and reduces nitrogen losses *via* leaching and gaseous emissions (Wang P. et al., 2021; Qin et al., 2024). Given the environmental and economic issues associated with excessive nitrogen fertilization, understanding and implementing BNI mechanisms can offer useful options for sustainable agriculture. Nitrification is a two-step microbial process wherein ammonia-oxidizing bacteria (AOB) and archaea (AOA) oxidize ammonium to nitrite (NO2^-^), followed by the conversion of nitrite to nitrate by nitrite-oxidizing bacteria (NOB) (Tyagi et al., 2022; Ayiti and Babalola, 2022). The resultant nitrate has significant mobility in soil, making it prone to leaching and potentially contaminating groundwater and exacerbating eutrophication. Nitrification produces nitrous oxide (N_2_O), a potent greenhouse gas. BNI mitigates environmental concerns by suppressing nitrifier activity, while maintaining nitrogen in a form readily accessible to plants, hence enhancing nitrogen retention in soils (Purswani and Llorente, 2021). Numerous plant species, particularly those in grasslands and agroecosystems, have been identified as sources of chemicals that hinder biological nitrification. Tropical grasses such as Brachiaria, sorghum and rice exhibit significant BNI potential. Brachiaria species emit hydrophobic chemicals as brachialactone, which selectively block ammonia monooxygenase (AMO), the essential enzyme in ammonia oxidation (Lata et al., 2022; Sadhukhan et al., 2022). Likewise, sorghum roots produce sorgoleone, a quinone-derived molecule known for its potent nitrification inhibitory properties (Wang W. et al., 2021). Acquiring a comprehensive grasp of these natural inhibitors and their mechanisms is essential for formulating agricultural practices centered on BNI. The genetic basis of BNI is an expanding area of study, concentrating on the identification of genes and metabolic pathways implicated in the synthesis and secretion of nitrification inhibitors (Wang X. et al., 2021; Ghatak et al., 2023). Recent progress in functional genomics and molecular biology has enabled the characterization of BNI-related genes in model plants and crops. Tools such as RNA sequencing and genome-wide association studies have enabled researchers to analyze gene activity and regulatory networks associated with key agronomic traits (Hirani et al., 2014; Kumar et al., 2016). In rice, wheat and maize, functional genomics has facilitated the identification of genes that encode TFs, protein kinases and signaling molecules that negatively control growth or stress responses (Vij and Tyagi, 2007). Once identified, these genes can be targeted as pharmacological inhibitors to augment desirable features. In wheat, functional genomics research has elucidated the role of DELLA proteins in gibberellin signaling, demonstrating that modifying these biological inhibitors has improved plant height and grain yield under semi-dwarf conditions (Sokolowska et al., 2025). Likewise, in rice, the identification of inhibitors in hormone pathways, such as abscisic acid (ABA) and salicylic acid (SA), has created opportunities to precisely modulate stress responses without hindering growth (Verma et al., 2016). Breeding projects are already incorporating BNI characteristics into high-yield cereal varieties to enhance nitrogen retention and reduce reliance on synthetic nitrification inhibitors such as dicyandiamide (DCD) and nitrapyrin (Saud et al., 2022). Furthermore, soil microbial populations are crucial for the efficacy of BNI. The diversity of nitrifier populations and their interactions with other soil microorganisms substantially influence the efficacy of nitrification suppression. Soil organic matter content, pH, temperature, and moisture levels influence BNI activity (Sadhukhan et al., 2022; Bozal-Leorri et al., 2022). Field studies indicate that crops exhibiting BNI activity can diminish nitrogen losses, enhance crop yields and foster long-term soil health. However, for BNI based techniques to gain widespread acceptance, further study is essential to comprehend the long-term impacts of BNI compounds on soil microbial populations and ecosystem functioning (Saud et al., 2022; Gawdiya and Kumar, 2025). The Hierarchical representation of plant intake that affects NUE are shown in Figure 2.

## 3 QTLs related with NUE

The quantitative structure of NUE and its strong sensitivity to environmental fluctuations have limited the effectiveness of traditional breeding methods for its improvement. To overcome these constraints, molecular breeding approaches such as QTL mapping have been utilized to elucidate the genetic foundation of NUE and pinpoint critical genomic areas linked to this characteristic (Ishfaq et al., 2023). QTLs are genomic regions that show statistical association with variation in a given characteristic. Identifying QTLs linked to NUE elucidates the genetic mechanisms involved and aids in the development of molecular markers for MAS (Ishfaq et al., 2023). Over the past 2 decades, many QTLs and candidate genes linked to NUE and its component traits have been identified in major cereal crops, such as rice (Cho et al., 2007), wheat (Fan et al., 2018) and maize (Sanchez et al., 2023). These QTLs affect characteristics including root architecture, nitrogen transporter activity, nitrate absorption efficiency and biomass production under varying nitrogen conditions. In this context, a study was conducted in which a recombinant inbred line (RIL) population comprising 166 F_8_ lines was established and utilized for QTL mapping in rice. The investigation identified 20 single-locus QTL (S-QTLs) and 58 epistatic quantitative trait locus pairs (E-QTLs). These QTLs showed strong associations with key agronomic and physiological traits, including nitrogen concentration in grain and straw, shoot nitrogen content, harvest index, grain yield, straw yield and physiological nitrogen use efficiency (PNUE) (Cho et al., 2007). The large number of single-locus and epistatic QTLs identified in the RIL population reflects the complex, polygenic nature of NUE and yield traits in rice (Wang et al., 2025). Additionally, advanced F_8_ lines possess high homozygosity, enabling precise phenotypic expression and improved QTL detection. Nitrogen-related traits involve multiple physiological processes such as uptake, assimilation and remobilization, which are governed by interacting genes, leading to significant epistasis. The relatively large population size enhanced recombination and genome coverage, facilitating the identification of multiple QTLs. Additionally, genetic correlations among traits such as grain yield, harvest index and nitrogen content contributed to the co-localization of QTLs (Jia et al., 2025). Furthermore, in rice, the QTL *qNUE-6* has been linked with enhanced root length and nitrogen uptake efficiency (Yang et al*.,* 2017). Consistent with this, earlier studies conducted during 2006 and 2007 identified five and six QTLs respectively, linked to NUE related trait across multiple chromosome including 1, 2, 3, 4, 6, 7, 9, 10 and 11. Additionally, four key regions, namely, G393-C922 on chromosome 1, RM232-C63 on chromosome 3, G235-G102 on chromosome 4 and RG678-R1440 on chromosome seven were consistently associated with NUE-related traits, highlighting their potential importance for improving NUE in rice (Wei et al., 2012). Supporting these findings, an association analysis was conducted using a population of 184 rice cultivar genotypes with 157 genome-wide SSR markers identified eight marker loci significantly correlated with NUE related traits. Among these, two QTLs located at RM5639 and RM3628 were linked to important NUE associated genes *GS1;2* and *AspAt3*, respectively which further emphasizing the genetic basis of NUE and its potential for target improvement (Zhou et al., 2017; Liu et al., 2022). Building on this, a novel locus, RM5748 was identified where Kompetitive allele specific PCR (KASP) markers were developed based on single nucleotide polymorphisms (SNPs), enabling more precise marker-assisted selection for NUE traits (Liu et al., 2016). Similar advances have been reported in other cereal crops, highlighting the conserved genetic control of NUE. In wheat, QTLs located on chromosomes 2A, 4D and 6B have been associated with enhanced nitrogen remobilization and increased grain protein content (Fan et al., 2018; Zhang et al., 2019). Similarly, in maize, QTLs such as *aco5* and *cdpk3* regulate nitrogen uptake and utilization, thereby influencing grain yield under low-nitrogen conditions (Sanchez et al., 2023). For additional QTLs related to NUE, refer to Supplementary Table 1. Additionally, recent developments in high-throughput genotyping and GWAS have markedly accelerated the identification of QTLs associated with NUE (Phan et al., 2023). Through the functional characterization of candidate genes linked to these QTLs, researchers have acquired significant insights into the principal regulatory networks governing nitrogen metabolism. Genes encoding nitrate transporters (from the NRT1 and NRT2 families), GS and GOGAT have been discovered within NUE-related QTL areas, highlighting their significance in nitrogen absorption processes in wheat (Zhang P. et al., 2023). To successfully integrate NUE QTLs into breeding programs, it is crucial to validate them across diverse genetic backgrounds and environmental conditions (Khan et al., 2024).

## 4 Transcription factors

A comprehensive understanding of the transcriptional regulatory networks governing NUE are essential for developing agricultural cultivars that exhibit enhanced nitrogen efficiency and reduced dependence on nitrogen fertilizers (Gao et al., 2022a). Moreover, TFs are proteins that attach to specific DNA sequences within the promoters of target genes, thereby modulating their transcriptional activity. Numerous transcription factor families have been found in plants many of which play a key role in nutrition signaling and regulation. Numerous families of TFs are implicated in nitrogen signaling that governs NUE (Liu et al., 1999). The classes are categorized into the NAC, MYB, bZIP, WRKY, AP2/ERF and NLP (Nodule Inception-like Protein) families (Bozal-Leorri et al., 2022). Each of these transcription factor families contributes to the diverse facets of nitrogen metabolism and homeostasis. TFs involved in regulating NUE in crops are presented in Table 1. This table enlists key TFs, such as Nodule Inception-like Proteins (NLPs), MYB, NAC, basic leucine zipper (bZIP) and WRKY families that are involved in the regulation of nitrogen uptake, remobilization and metabolism across major crops like rice, wheat, maize and barley. Each TF is associated with specific genes, corresponding nitrogen-related traits and relevant literature references. These transcription factors operate within complex regulatory networks, responding to internal nitrogen status and external nitrogen availability, while also interacting with other signaling pathways such as carbon metabolism and hormonal regulation (Sathee et al., 2025). TFs regulate the expression of genes associated with nitrate and ammonium transporters, NR, glutamine synthetase, glutamate synthase and other metabolic enzymes, thereby orchestrating nitrogen uptake and use under diverse environmental conditions (Zhang H. et al., 2023). The expression of these transporters is tightly regulated by multiple TFs. NLP (NIN-like protein) TFs, especially NLP7 in *Arabidopsis* has been recognized as a pivotal regulator of nitrogen-responsive gene expression (Wang M. et al., 2024). NLP7 interacts with nitrate-responsive cis-elements in the promoters of nitrate inducible genes and modulates a wide array of genes associated with nitrate absorption and assimilation. In nitrate abundant circumstances, NLP7 is activated and relocated to the nucleus, where it interacts with its target DNA sequences to commence transcription. Both loss-of-function and mutant variants of NLP7 exhibit compromised nitrate absorption and diminished growth, underscoring its critical role in NUE (Wang Y. et al., 2024). The TCP family of TFs, including TCP20, plays a role in root growth and nitrogen foraging responses. TCP20 interacts with NLP6 and NLP7 to modulate the expression of nitrate-responsive genes (Kumar et al., 2018). The MYB family constitutes another significant category of TFs linked to NUE. In rice, *OsMYB305* has been shown to improve nitrate absorption and NUE by upregulating genes encoding NR and glutamine synthetase. The overexpression of *OsMYB305* in rice enhanced biomass and grain yield under low nitrogen conditions, positioning it as a promising option for genetic enhancement of NUE (Wang D. et al., 2020). Furthermore, DOF (DNA-binding with one finger) TFs serve as essential regulators of nitrogen metabolism. In maize, *ZmDOF1* is associated with the regulation of carbon and nitrogen metabolism. The overexpression of *ZmDOF1* in transgenic plants increases the expression of genes associated with nitrogen assimilation, including NR and glutamate synthase, thereby enhancing nitrogen uptake and utilization (Kurai et al., 2011; Zuluaga and Sonnante, 2019). Moreover, DOF1 directly modulates phosphoenolpyruvate carboxylase (PEPC), a crucial enzyme that integrates carbon and nitrogen metabolism, hence underscoring its function in regulating the C:N balance in plants (Babele et al., 2023; Liu et al., 2025). Moreover, many NAC TFs have been linked to nutritional responses, particularly nitrogen. In rice, *OsNAC42* regulates nitrogen deprivation responses by influencing the expression of genes associated with nitrogen transport and remobilization (Tang et al., 2019). These TFs either activate or inhibit the transcription of downstream genes that influence nitrogen metabolism and redistribution, especially in nutrient-deficient environments. Conversely, the bZIP family constitutes another category of TFs associated with NUE (Hossain et al*.,* 2016). BZIP TFs participate in food and hormone signaling pathways (Hasegawa et al., 2021). Furthermore, WRKY TFs, initially recognized for their function in plant defense and stress responses, have also been associated with nutrition control. Recent findings indicate that WRKY TFs can affect nitrogen absorption and assimilation by regulating the expression of NRT and nitrogen metabolic genes (Khong et al., 2008). *OsWRKY45* governs nitrogen remobilization in rice during senescence, a vital stage for nitrogen recycling and grain development. WRKY TFs enhance NUE and agricultural output by regulating the timing and efficacy of nitrogen remobilization (Khong et al., 2008; Kan et al., 2015). Additionally, the interaction between nitrogen signaling and hormonal pathways encompasses TFs. Auxin, cytokinin, abscisic acid, and ethylene signaling pathways engage with nitrogen metabolism at various levels. TFs, including auxin response factors (ARFs) and the cytokinin response regulator ARR1, regulates gene expression in response to nitrogen and hormonal signals, thereby integrating environmental stimuli to optimize nitrogen acquisition and use (Ohri et al., 2015; Parwez et al., 2022). Furthermore, spontaneous variation in transcription factor genes across crop genotypes have been investigated to uncover variants linked to elevated NUE. GWAS and QTL mapping have identified multiple TF genes associated with NUE-related traits, including nitrogen uptake efficiency, NUtE, root architecture, and grain yield (Shi et al., 2022). Variations in *OsNLP3* and *OsDOF18* have been linked to nitrogen response features in rice (Zhang B. et al., 2022). These findings offer molecular markers for marker-assisted selection and the breeding of NUE-enhanced cultivars.

**TABLE 1:** TFs regulating nitrogen metabolism in different crop plants.

Gene	Trait	Crop name	References
*NLP1*	Nitrogen uptake	Rice	Jagadhesan et al. (2020)
*NLP3*			
*NLP4*			
*NLP5*			
*OsNLP5*			
*OsNRT2.1*			
*OsNRT2.2*			
*OsNRT2.3a*			
*NLP2*			Chan (2023)
*NLP7*			
*OsNLP1*			Alfatih et al. (2020)
*OsNLP3*			Zhang Z. S. et al. (2022)
*OsNLP4*			Wu et al. (2021)
*TaNLP1*		Wheat	Konishi and Yanagisawa (2013)
*TaNLP2*			
*NLP7*			Kumar et al. (2018)
*TaNLP1*			
*TaNLP2*			
*TaNLP4*			Mishra et al. (2025)
*TaNLP5*			
*TaNLP17*			
*TaNLP18*			
*TaNLP2*			
*AtNLP8*			
*AtNLP6*			
*AtNLP7*			
*ZmNLP3*		Maize	Ge et al. (2018)
*ZmNLP5*			
*ZmNLP9*			
*ZmNLP4*			
*ZmNLP6*			
*ZmNLP8*			
*ZmNLP15*			Jiang et al. (2018)
*ZmNLP6*			Wang D. et al. (2020)
*hvnlp2-1*		Barley	Gao et al. (2022b)
*hvnlp2-2*			
*hvnlp2-3*			
*OsNLP4*	Nitrate assimilation	Rice	Wang M. et al. (2020)
*OsGOGAT1*			Zhang Y. et al. (2023)
*OsGOGAT2*			
*OsNIA2*			
*OsMYB55*	Nitrogen remobilization	Rice	Beatty and Good (2018)
*MYB61*			Gao et al. (2020)
*OsMYB305*			Wang Y. et al. (2020)
*OsMYB61*			Gao et al. (2020)
*OsMYB102*			Kumari et al. (2021)
*OsMYB59*			Islam et al. (2021)
*HvMYB1*		Barley	Ma and Wang (2025)
*OsNAC42*	Nitrate uptake	Rice	Tang et al. (2019)
*TaNAC2-5A*			He et al. (2015)
*OsSNAC1*			Qi et al. (2023)
*OsNAC3*			Kumari et al. (2021)
*OsNRT1*			Zhang Y. et al. (2023)
*OsNRT1.1B*			
*OsNPF2.4*			
*OsbZIP46*	Nitrogen uptake	Rice	Hossain et al. (2016)
*OUR1/OsbZIP1*			Hasegawa et al. (2021)
*WRKY45*	Nitrogen metabolism	Rice	Khong et al. (2008)
*WRKY69*			Kan et al. (2015)
*OsWRKY23*			Zhang et al. (2025)
*HvWRKY23*		Barley	Ma and Wang (2025)
*NRT1*	Nitrate transporter	Rice	Nazish et al. (2022)
*NRT2*			

## 5 miRNA involved in different aspects of NUE

miRNAs are diminutive non-coding RNAs that play a crucial roles in post-transcriptional control by targeting mRNAs for either destruction or translational suppression. Regarding NUE, miRNAs can modulate specific physiological and molecular processes related to nitrogen uptake, assimilation, translocation and remobilization. Understanding the roles of miRNAs in NUE may lay the foundation for innovative approaches to improve agricultural NUE and reduce dependence on nitrogen fertilizers (Zhang et al., 2025). For instance, a study integrated microRNA sequencing of panicle tissues at the booting stage with microarray profiling of root and shoot tissues at the seedling stage across N-efficient and non-efficient rice genotypes under N^+^ and N^−^ conditions to elucidate NUE regulation (Barbadikar et al., 2025). The analysis revealed that N-efficient genotypes exhibited upregulation of miR2118o, miR1442 and miR5149, along with downregulation of miR164, miR2867 and miR171i, with consistent expression patterns of miR1859 (downregulated) and miR1441 and miR3979-5p (upregulated). These expression changes were associated with key pathways involving nodulin genes, receptor kinases, auxin-related genes and transporters, while Polycomb-associated genes were suppressed under low nitrogen conditions (Barbadikar et al., 2025). Thus, the findings demonstrate that NUE is governed by coordinated microRNA–mRNA interactions and metabolic adjustments, highlighting promising candidate targets for improving nitrogen efficiency in rice. Some miRNAs are used to enhance traits related to NUE are presented in Table 2.

**TABLE 2:** List of miRNAs and associated genes involved in nitrogen metabolism in various crops.

S. No.	miRNAs name	Crop	Traits	References
1	miR167	Wheat	Nitrogen uptake	Das and Sathee (2023)
2	miR393	Rice		Kong et al. (2021)
3	miR169	Maize		Zuluaga and Sonnante (2019)
4	miR1214			
5	miR2199			
6	miR398			
7	miR408			
8	miR827			
9	miR2118o	Rice		Barbadikar et al. (2025)
10	miR1442			
11	miR5149			
12	miR164			
13	miR2867			
14	miR171i			
15	miR1859			
16	miR1441			
17	miR3979-5p			
18	miR5149			
19	miR169o			Yu et al. (2018)
20	Osa-miR528			Zhao et al. (2022)
21	OsGS1	Rice	Nitrogen assimilation	Kong et al. (2021)
22	miR827			Zuluaga and Sonnante (2019)
23	miR528			Yuan et al. (2015)
24	miR156			Gao et al. (2020)
25	miR399		Nitrogen translocation	Mazahar and Umar (2025)
26	miR398			Kumar (2014)
27	miR166			Iwamoto and Tagiri (2016)
28	miR156	Wheat	Nitrogen remobilization and senescence	Niazi et al. (2023)
29	miR172	Rice		Das et al. (2023)

This table summarizes miRNAs reported in major crops like rice, wheat and maize that regulate different aspects of nitrogen metabolism. The associated crop, the specific nitrogen-related trait (e.g., uptake, assimilation, translocation, or remobilization) and the corresponding literature references are provided.

## 6 CRISPR/Cas9

**FIGURE 3:**
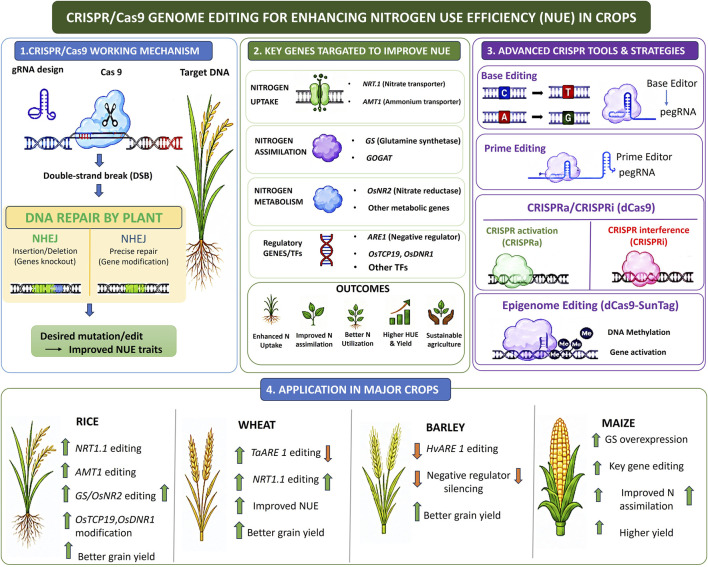
Schematic representation of the CRISPR/Cas9 genome editing system for NUE traits.

The advent of CRISPR/Cas9 based genome editing has generated promising prospects for improving NUE by precise alterations of genes associated with nitrogen uptake, assimilation and metabolism. This innovative approach, derived from the bacterial adaptive immune system facilitates precise alterations in plant genomes by generating specific mutations in genes involved in nitrogen metabolism (Mudiyanselage, 2021; Elsharawy and Refat, 2023; Biradar et al., 2024). The technique involves designing a guide RNA (gRNA) targeting the target gene, while the Cas9 endonuclease induces double-strand breaks (DSBs) at the designated site. The plant subsequently employs its inherent repair mechanisms, either non-homologous end joining (NHEJ) or homology-directed repair (HDR), to induce mutations that may improve NUE features (Aksoy et al., 2022; Pal et al., 2023). Numerous key genes have been identified as major regulators of NUE and the CRISPR/Cas9 genome editing system has emerged as a powerful tool to precisely modify these genes, thereby enhancing nitrogen uptake and utilization (Liu et al., 2023). This technology was first successfully demonstrated in 2013, when it was used to target multiple genes in rice and wheat (Shan et al., 2013), paving the way for its widespread application in crop improvement. Since then, several nitrogen transporter genes have been edited to improve NUE, including NRT1.1, whose modification has significantly enhanced nitrate uptake efficiency in crops such as rice and wheat. In a similar context, the AMT1 gene, which plays a crucial role in ammonium acquisition, has also been targeted to improve ammonium uptake efficiency (Hou et al., 2021; Kumar et al., 2024). Building on these advances, CRISPR/Cas9 has further been utilized to manipulate key enzymes involved in nitrogen assimilation, particularly GS, resulting in improved NUE in both maize (Zhu et al., 2016) and rice (Lebedev et al., 2021). Moreover, the technology has been extended to functional characterization of genes such as *OsHKT1;4* in rice, providing insights into their roles in NUE (Mohammed, 2018). In addition to structural and enzymatic genes, transcription factors (TFs) that regulate nitrogen-responsive pathways are increasingly being recognized as promising targets for CRISPR/Cas9-mediated modification (Wu et al., 2024), further broadening the scope of NUE improvement strategies. Collectively, these advancements highlight the significant potential of CRISPR/Cas9 in enhancing NUE across diverse crop species. This is particularly important for crops like rice, which require substantial nitrogen inputs for optimal productivity (Hou et al., 2021). In this regard, the rice ARE1 gene homolog has been identified as a viable target, and its downregulation has been shown to improve NUE in barley (Wang et al., 2018). Therefore, CRISPR/Cas9-mediated gene silencing or downregulation offers an effective and targeted strategy for improving NUE. However, it is noteworthy that most genetic improvement efforts to date have predominantly focused on gene overexpression approaches. A schematic representation of CRISPR/Cas9-mediated genome editing is presented in Figure 3.

**FIGURE 4:**
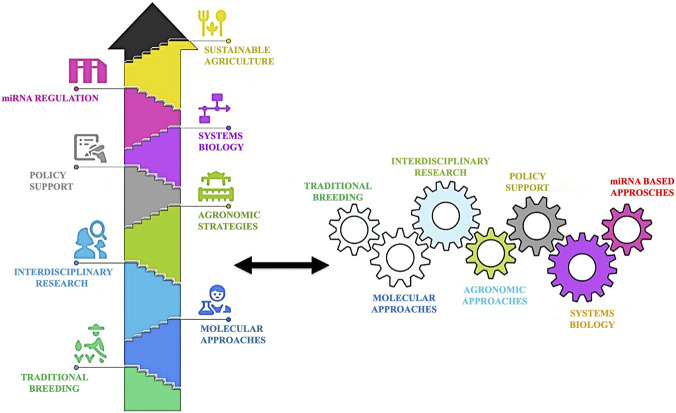
A conceptual framework showing interdisciplinary approaches for improving NUE. Traditional breeding, molecular tools, systems biology interact together to achieve sustainable agriculture and long-term productivity.

Moreover, it is imperative to rigorously evaluate the field performance of CRISPR edited crops under diverse environmental conditions to ensure consistent and improvements in NUE. In this regard, significant progress has been made through targeted genome editing approaches. For instance, a study identified three *TaARE1* homologs gene in the high-performing Chinese winter wheat variety ZhengMai 7698 by employing CRISPR/Cas9-mediated targeted mutagenesis, researchers successfully generated numerous transgene free mutant lines carrying either partial or triple null *TaARE1* alleles, which demonstrated promising potential for improving NUE (Zhang Y. et al., 2021). Building on such advancements, the application of CRISPR/Cas9 was extended to other cereals, with its first successful use in barley reported by Lawrenson et al. (2015). Subsequently, further research focused on the barley *Abnormal* *cytokinin response1 repressor 1* (*HvARE1*) gene which had been identified as a candidate regulator of NUE through genome-wide association studies. This study integrated the analysis of natural genetic variation with CRISPR/Cas9-based gene editing to validate the functional role of *HvARE1*, thereby highlighting the growing significance of genome editing tools in enhancing NUE across major cereal crops (Karunarathne et al., 2022). Furthermore, *OsTOND1* is a gene linked to a QTL conferring tolerance to nitrogen (N) deficiency in rice. It encodes a thaumatin-like protein and is essential for primary root extension under low-nitrogen conditions. *OsTOND1* has been identified in some indica rice varieties but it is absent in the japonica types examined so far, indicating subspecies-specific genetic variation that may influence NUE (Zhang et al., 2015). Building on this genetic diversity, studies have shown that concurrent overexpression of multiple nitrogen related genes can significantly enhance NUtE in rice. For instance, transgenic lines overexpressing either *OsNRT2.1* or *OsNAR2.1* individually exhibited a 10% increase in 15 NO_3_^−^ inflow rate, a 20% improvement in agronomic NUE and a 30% improvement in nitrogen recovery efficiency compared to wild-type plants demonstrating the potential of gene-based strategies for improving nitrogen utilization. Conversely, lines co-overexpressing both *OsNRT2.1* and *OsNAR2.1* exhibited even more significant enhancements, with increases of 40%, 50% and 60% in the corresponding parameters (Hou et al., 2021). Furthermore, CRISPR/Cas9 was utilized to modify *OsTCP19* and *OsDNR1*, while a CRISPR/Cas9 based base editor was applied for the exact alteration of *OsNRT1.1B*. Prime editing was utilized to target *OsNR2*. Likewise, *OsNRT1.1B*, which encodes a nitrate transporter, contains a significantly divergent nonsynonymous SNP (980C>T) that differentiates *japonica* from *indica* rice subpopulations. This SNP correlates with enhanced NUE in *indica* cultivars (Hu et al., 2015). A cytidine base editor was utilized to incorporate the 980T allele into the japonica cultivar JXY1. *OsNRT1.1B* augments the expression of *OsNR2*, which encodes an NR. In indica rice cultivars, a crucial amino acid substitution of arginine at position 783 (Arg783) due to an SNP (2335T>A) markedly enhances the enzymatic activity of *OsNR2* (Gao et al., 2019). Furthermore, this study examined alternative genome editing strategies targeting key positive and negative regulatory regions. CRISPR based systems that facilitate transcriptional activation or repression via catalytically inactive Cas9 (dCas9) are referred to as CRISPR activation (CRISPRa) and CRISPR interference (CRISPRi), respectively. The dCas9-SunTag technology facilitates precise DNA methylation and gene activation in plants. Genome editing can be utilized to alter negative regulators of nutrient signaling, thereby improving nutrient absorption and stress responses, especially in resource-constrained contexts (Hu et al., 2015). Promoter engineering using CRISPR/dCas9 systems in combination with cytosine and adenine base editors or prime editing provide an accurate method for executing targeted genomic modifications. Moreover, the incorporation of transcriptional activators and repressors inside the CRISPR/dCas9 system facilitates the precise overexpression of target genes, providing enhanced flexibility and accuracy in gene regulation (Mytton and Skøt, 1993; Lebedev et al., 2021; Jain et al., 2023; Sathee et al., 2023). A conceptual framework highlighting the necessity of multifaceted, interdisciplinary strategies to achieve sustainable agriculture, focusing on enhancing NUE, as shown in Figure 4.

## 7 Future prospects

Traditional breeding is crucial for improving NUE in cereal crops. At the same time, the integration of molecular approaches such as MAS, GWAS, GS and genome editing (CRISPR/Cas9) could significantly accelerate advancements. The advancement of NUE enhancement will rely on interdisciplinary research integrating genetics, bioinformatics and agronomic breakthroughs to produce resilient, high-yield crops that necessitate reduced nitrogen input. Moreover, cooperation among plant breeders, molecular biologists and agronomists will be essential to translate laboratory advances into viable field applications. Integrating traditional knowledge with advanced biotechnological innovations enables the development of crop types that thrive in low-nitrogen conditions while maintaining yield. In conjunction with breeding programs, agronomic approaches such as precision farming, efficient nutrient management and sustainable soil health strategies will collaboratively enhance NUE. Furthermore, it is imperative for governments and policymakers to support research initiatives, infrastructure development and knowledge dissemination to facilitate the widespread adoption of NUE-enhanced agricultural varieties. Furthermore, future investigations on NUE should prioritize integrating genetic and agronomic methodologies to provide comprehensive answers. Advancements in systems biology, coupled with genome-editing tools such as CRISPR/Cas9, can expedite the development of nitrogen-efficient crop varieties and improve fertilizer application practices. Future research priorities should include fine-mapping of NUE-associated QTLs, investigating gene interactions and developing breeding techniques that integrate various traits to achieve a balance among nitrogen efficiency, high yield and quality. Recent breakthroughs in genetic engineering and genome editing, notably utilizing CRISPR/Cas9 technology, permit precise alterations in miRNA expression to improve NUE. Through the overexpression or suppression of certain miRNAs associated with NUE, it may be feasible to cultivate crops that have enhanced nitrogen uptake, assimilation and remobilization abilities. In conclusion, miRNAs function as critical regulators of NUtE, influencing nitrogen uptake, assimilation, translocation and remobilization. By using miRNA-based approaches, researchers can develop nitrogen-efficient crops that support sustainable agricultural practices. Additional research is required to elucidate the relationships between miRNAs and other regulatory networks, which will help formulate novel techniques to enhance cereal crops.

## 8 Conclusion

NUE in cereal crops represents a central challenge and opportunity for achieving sustainable agricultural productivity, food security and environmental protection. This review demonstrates that NUE is a highly complex and integrative trait governed by tightly coordinated physiological, biochemical and molecular processes, including nitrogen uptake, assimilation, remobilization and utilization. Key components such as NUpE, NUtE and NRE define NUE are strongly influenced by root architecture, transporter activity, canopy structure and photosynthetic performance. Additionally, at the molecular level, NUE is regulated by an extensive regulatory network of TFs, including NLP, MYB, NAC, bZIP, WRKY, DOF and TCP families, which integrate nitrogen signaling with carbon metabolism and hormonal pathways. These TFs orchestrate the expression of genes involved in nitrate and ammonium transport, nitrogen assimilation enzymes and metabolic coordination, thereby controlling plant performance under variable nitrogen conditions. In parallel, post-transcriptional regulation via miRNAs adds an additional layer of complexity in fine-tuning nitrogen responses. However, recent advances in genomics and biotechnology have significantly accelerated NUE improvement. QTL mapping, GWAS and genomic selection have enabled the identification of key genomic regions and candidate genes associated with nitrogen efficiency traits. Most notably, CRISPR/Cas9 and related genome editing technologies such as base editing, prime editing, CRISPRa/i have emerged as transformative tools for precise manipulation of nitrogen-related genes, including transporters (NRTs, AMTs), assimilation enzymes (GS, NR) and key regulatory TFs. These technologies allow targeted improvement of nitrogen acquisition, assimilation efficiency and remobilization, leading to enhanced yield stability under low-input systems. Furthermore, integrating BNI improved nitrogen management strategies and plant-microbe interactions provide an additional sustainable dimension to NUE enhancement by reducing nitrogen losses and improving soil nitrogen retention. So, the convergence of conventional breeding, molecular genetics, systems biology and advanced genome editing offers a powerful and sustainable framework for developing next-generation cereal cultivars with superior NUE. Such integrated strategies are essential to reduce dependency on synthetic nitrogen fertilizers, minimize environmental pollution and ensure stable grain yield and quality under changing climatic conditions. Collectively, improving NUE through multidisciplinary approaches represents a cornerstone for future climate-resilient and environmentally sustainable cereal-based agriculture.

## References

[B1] Aasfar A. Bargaz A. Yaakoubi K. Hilali A. Bennis I. Zeroual Y. (2021). Nitrogen fixing azotobacter species as potential soil biological enhancers for crop nutrition and yield stability. Front. Microbiol. 12, 628379. 10.3389/fmicb.2021.628379 33717018 PMC7947814

[B2] Agrama H. A. (2006). Application of molecular markers in breeding for nitrogen use efficiency. J. Crop Improv 15, 175–211. 10.1300/J411v15n01_09

[B3] Ahmad W. Bibi N. Sanwal M. Ahmed R. Jamil M. Kalsoom R. (2024). Cereal crops in the era of climate change: an overview. Environ. Clim. Plant Veget Growth, 609–630. 10.1007/978-3-031-69417-2_21

[B4] Akhtar K. Ain N. U. Prasad P. V. Naz M. Aslam M. M. Djalovic I. (2024). Physiological, molecular, and environmental insights into plant nitrogen uptake and metabolism under abiotic stresses. Plant Genome 17 (2), e20461. 10.1002/tpg2.20461 38797919 PMC12806923

[B5] Aksoy E. Yildirim K. Kavas M. Kayihan C. Yerlikaya B. A. Çalik I. (2022). General guidelines for CRISPR/Cas-based genome editing in plants. Mol. Biol. Rep. 49, 12151–12164. 10.1007/s11033-022-07655-0 36107373

[B6] Alfatih A. Wu J. Zhang Z. S. Xia J. Q. Jan S. U. Yu L. H. (2020). Rice NIN-LIKE PROTEIN 1 rapidly responds to nitrogen deficiency and improves yield and nitrogen use efficiency. J. Exp. Bot. 71, 6032–6042. 10.1093/jxb/eraa345 32585013

[B8] Ali M. F. Han R. Lin X. Wang D. (2025). Controlled-release nitrogen combined with ordinary nitrogen fertilizer improved nitrogen uptake and productivity of winter wheat. Front. Plant Sci. 15, 1504083. 10.3389/fpls.2024.1504083 39840365 PMC11747511

[B9] Alrajhi A. Alharbi S. Beecham S. Alotaibi F. (2024). Regulation of root growth and elongation in wheat. Front. Plant Sci. 15, 1397337. 10.3389/fpls.2024.1397337 38835859 PMC11148372

[B10] Andualem A. Wato T. Asfaw A. Urgi G. (2024). Improving primary nutrients (NPK) use efficiency for the sustainable production and productivity of cereal crops: a comprehensive review. J. Agric. Sustain Environ. 2997, 271X. 10.56556/jase.v3i1.833

[B11] Ayiti O. E. Babalola O. O. (2022). Factors influencing soil nitrification process and the effect on environment and health. Front. Sustain Food Syst. 6, 821994. 10.3389/fsufs.2022.821994

[B12] Babele P. K. Srivastava A. Selim K. A. Kumar A. (2023). Millet-inspired systems metabolic engineering of NUE in crops. Trends Biotechnol. 41, 701–713. 10.1016/j.tibtech.2022.09.004 36566140

[B13] Barbadikar K. M. Talapanti K. K. Bej S. Karre S. Kothandaraman H. Malathi S. (2025). Integrated omics analysis of rice gene expression profiles and microRNAs identifies crucial target genes for nitrogen use efficiency. Plant Physiol. Rep. 30, 379–399. 10.1007/s40502-024-00846-9

[B14] Barłóg P. Grzebisz W. Łukowiak R. (2022). Fertilizers and fertilization strategies mitigating soil factors constraining efficiency of nitrogen in plant production. Plants 11 (14), 1855. 10.3390/plants11141855 35890489 PMC9319167

[B15] Beatty P. H. Good A. G. (2018). “Improving nitrogen use efficiency in crop plants using biotechnology approaches,” in Engineering Nitrogen Utilization in Crop Plants (Cham: Springer), 15–35. 10.1007/978-3-319-92958-3_2

[B16] Bhardwaj E. Shukla R. Das S. (2021). Plant roots and mineral nutrition: an overview of molecular basis of uptake and regulation, and strategies to improve nutrient use efficiency (NUE). Plant Stress Biol. Strategies Trends, 131–184. 10.1007/978-981-15-9380-2_5

[B17] Biradar S. S. Patil M. K. Desai S. A. Singh S. K. Naik V. R. Lamani K. (2024). Nitrogen use efficiency in bread wheat: genetic variation and prospects for improvement. PLoS ONE 19, e0294755. 10.1371/journal.pone.0294755 38598487 PMC11006162

[B18] Bodner G. Mentler A. Keiblinger K. (2021). “Plant roots for sustainable soil structure management in cropping systems,” in The Root Systems in Sustainable Agricultural Intensification (Springer), 45–90.

[B19] Bozal-Leorri A. Subbarao G. V. Kishii M. Urmeneta L. Kommerell V. Karwat H. (2022). Biological nitrification inhibitor-trait enhances nitrogen uptake by suppressing nitrifier activity and improves ammonium assimilation in two elite wheat varieties. Front. Plant Sci. 13, 1034219. 10.3389/fpls.2022.1034219 36438125 PMC9695736

[B20] Buchner P. Hawkesford M. J. (2014). Complex phylogeny and gene expression patterns of members of the nitrate transporter 1/peptide transporter family (NPF) in wheat. J. Exp. Bot. 65, 5697–5710. 10.1093/jxb/eru231 24913625 PMC4176842

[B21] Burgess A. J. Masclaux-Daubresse C. Strittmatter G. Weber A. P. Taylor S. H. Harbinson J. (2023). Improving crop yield potential: underlying biological processes and future prospects. Food Energy Secur 12, e435. 10.1002/fes3.435 37035025 PMC10078444

[B22] Carbajal-Friedrich A. A. Burgess A. J. (2024). The role of the ideotype in future agricultural production. Front. Plant Physiol. 2, 1341617. 10.3389/fpls.2024.1341617

[B23] Carr T. W. Addo F. Palazzo A. Havlik P. Pérez-Guzmán K. Ali Z. (2024). Addressing future food demand in the Gambia: can increased crop productivity and climate change adaptation close the supply–demand gap? Food secur. 16, 691–704. 10.1007/s12571-024-01397-2 38770159 PMC11102352

[B24] Chan C. (2023). The distinct functions of nodule inception-like proteins in nitrate response. Plant Cell. 35, 1296–1297. 36781394 10.1093/plcell/koad038 PMC10118256

[B25] Cho Y. I. Jiang W. Chin J. H. Piao Z. Cho Y. G. McCouch S. R. (2007). Identification of QTLs associated with physiological nitrogen use efficiency in rice. Mol. Cells 23, 72–79. 10.1016/S1016-8478(23)07391-0 17464214

[B26] Ciampitti I. A. Lemaire G. (2022). From use efficiency to effective use of nitrogen: a dilemma for maize breeding improvement. Sci. Total Environ. 826, 154125. 10.1016/j.scitotenv.2022.154125 35219655

[B27] Das S. Sathee L. (2023). miRNA-mediated regulation of nitrogen response and nitrogen use efficiency of plants: the case of wheat. Physiol. Mol. Biol. Plants 29, 1371–1394. 10.1007/s12298-023-01338-6 38076770 PMC10709294

[B28] Dave K. Kumar A. Dave N. Jain M. Dhanda P. S. Yadav A. (2024). Climate change impacts on legume physiology and ecosystem dynamics: a multifaceted perspective. Sustainability 16, 6026. 10.3390/su16156026

[B29] Dixon L. E. Van Esse W. Hirsz D. Willemsen V. McKim S. M. (2022). Cereal architecture and its manipulation. Annu. Plant Rev. 5, 1–54. 10.1002/9781119312994.apr0771

[B30] Dollete D. Lumactud R. A. Carlyle C. N. Szczyglowski K. Hill B. Thilakarathna M. S. (2024). Effect of drought stress on symbiotic nitrogen fixation, soil nitrogen availability, and soil microbial diversity in forage legumes. Plant Soil 495, 445–467. 10.1007/s11104-023-06383-8

[B31] Driever S. M. Mossink L. Ocaña D. N. Kaiser E. (2023). A simple system for phenotyping of plant transpiration and stomatal conductance response to drought. Plant Sci. 329, 111626. 10.1016/j.plantsci.2023.111626 36738936

[B32] Dubey R. S. Srivastava R. K. Pessarakli M. (2021). “Physiological mechanisms of nitrogen absorption and assimilation in plants under stressful conditions,” in Handbook of Plant and Crop Physiology. Editor Pessarakli M. (Boca Raton: CRC Press), 579–616. 10.1201/9781003044065-34

[B33] Elrys A. S. Elnahal A. S. Abdo A. I. Desoky E. S. M. Selem E. Rady M. M. (2022). Traditional, modern, and molecular strategies for improving the efficiency of nitrogen use in crops for sustainable agriculture: a fresh look at an old issue. J. Soil Sci. Plant Nutr. 22, 3130–3156. 10.1007/s42729-022-00915-y

[B34] Elsharawy H. Refat M. (2023). CRISPR/Cas9 genome editing in wheat: enhancing quality and productivity for global food security—A review. Funct. Integr. Genomics 23, 265. 10.1007/s10142-023-00978-2 37541970

[B35] Esmaeilzadeh-Salestani K. Samandari-Bahraseman M. R. Tohidfar M. Khaleghdoust B. Keres I. Mõttus A. (2023). Expression of AMT1AMT1;1 and AMT2;1 is stimulated by mineral nitrogen and reproductive growth stage in barley under field conditions. J. Plant Nutr. 46, 1246–1258. 10.1080/01904167.2023.2197954

[B36] Fan X. Zhang W. Zhang N. Chen M. Zheng S. Zhao C. (2018). Identification of QTL regions for seedling root traits and their effect on nitrogen use efficiency in wheat (*Triticum aestivum* L.). Theor. Appl. Genet. 131, 2677–2698. 10.1007/s00122-018-3189-x 30255337

[B37] Fang X. Z. Fang S. Q. Ye Z. Q. Liu D. Zhao K. L. Jin C. W. (2021). NRT1.1NRT1.1 dual-affinity nitrate transport/signaling and its roles in plant abiotic stress resistance. Front. Plant Sci. 12, 715694. 10.3389/fpls.2021.715694 34497626 PMC8420879

[B38] Fiaz S. Wang X. Khan S. A. Ahmar S. Noor M. A. Riaz A. (2021). Novel plant breeding techniques to advance nitrogen use efficiency in rice: a review. GM Crops Food 12, 627–646. 10.1080/21645698.2021.1971506 34034628 PMC9208628

[B39] Fortunato S. Nigro D. Lasorella C. Marcotuli I. Gadaleta A. de Pinto M. C. (2023). The role of glutamine synthetase (GS) and glutamate synthase (GOGAT) in the improvement of nitrogen use efficiency in cereals. Biomolecules 13 (12), 1771. 10.3390/biom13121771 38136642 PMC10742212

[B40] Fradgley N. S. Bentley A. R. Swarbreck S. M. (2021). Defining the physiological determinants of low nitrogen requirement in wheat. Biochem. Soc. Trans. 49, 609–616. 10.1042/bst20200282 33769462 PMC8106490

[B41] Freschet G. T. Pagès L. Iversen C. M. Comas L. H. Rewald B. Roumet C. (2021). A starting guide to root ecology: strengthening ecological concepts and standardizing root classification, sampling, processing, and trait measurements. New Phytol. 232, 973–1122. 10.1111/nph.17572 34608637 PMC8518129

[B42] Gao Z. Wang Y. Chen G. Zhang A. Yang S. Shang L. (2019). The indica nitrate reductase gene OsNR2 allele enhances rice yield potential and nitrogen use efficiency. Nat. Commun. 10, 5207. 10.1038/s41467-019-13110-8 31729387 PMC6858341

[B43] Gao Y. Xu Z. Zhang L. Li S. Wang S. Yang H. (2020). MYB61 is regulated by GRF4 and promotes nitrogen utilization and biomass production in rice. Nat. Commun. 11, 5219. 10.1038/s41467-020-19019-x 33060584 PMC7566476

[B44] Gao Y. Qi S. Wang Y. (2022a). Nitrate signaling and use efficiency in crops. Plant Commun. 3, 100305. 10.1016/j.xplc.2022.100353 35754172 PMC9483113

[B45] Gao Y. Quan S. Lyu B. Tian T. Liu Z. Nie Z. (2022b). Barley transcription factor HvNLP2 mediates nitrate signaling and affects nitrogen use efficiency. J. Exp. Bot. 73, 770–783. 10.1093/jxb/erab245 34050753

[B46] Gara T. W. Rahimzadeh-Bajgiran P. Darvishzadeh R. (2021). Forest leaf mass per area (LMA) through the eye of optical remote sensing: a review and future outlook. Remote Sens. 13, 3352. 10.3390/rs13173352

[B47] Garnett T. Conn V. Kaiser B. N. (2009). Root based approaches to improving nitrogen use efficiency in plants. Plant, Cell and Environ. 32, 1272–1283. 10.1111/j.1365-3040.2009.02011.x 19558408

[B48] Gawdiya S. Kumar D. (2025). Biological nitrification inhibition enabled climate-smart nitrogen efficient genotypes for today and tomorrow. Indian J. Fertilisers 21, 112–119.

[B49] Ge M. Liu Y. Jiang L. Wang Y. Lv Y. Zhou L. (2018). Genome-wide analysis of maize NLP transcription factor family revealed the roles in nitrogen response. Plant Growth Regul. 84, 95–105. 10.1007/s10725-017-0324-x

[B50] Ghatak A. Chaturvedi P. Waldherr S. Subbarao G. V. Weckwerth W. (2023). PANOMICS at the interface of root–soil microbiome and BNI. Trends Plant Sci. 28, 106–122. 10.1016/j.tplants.2022.08.016 36229336

[B51] Gooding M. J. Shewry P. R. (2022). Wheat: Environment, Food and Health. Hoboken, NJ, USA: John Wiley and Sons.

[B52] Govindasamy P. Muthusamy S. K. Bagavathiannan M. Mowrer J. Jagannadham P. T. K. Maity A. (2023). Nitrogen use efficiency—A key to enhance crop productivity under a changing climate. Front. Plant Sci. 14, 1121073. 10.3389/fpls.2023.1121073 37143873 PMC10151540

[B53] Grzyb A. Wolna-Maruwka A. Niewiadomska A. (2021). The significance of microbial transformation of nitrogen compounds in the light of integrated crop management. Agronomy 11, 1415. 10.3390/agronomy11071415

[B54] Gupta N. K. Shavrukov Y. Singhal R. K. Borisjuk N. (2023). Multiple Abiotic Stress Tolerances in Higher Plants: Addressing the Growing Challenges (Boca Raton, FL, USA: CRC Press).

[B55] Hasan A. Tabassum B. Hashim M. Khan N. (2024). Role of plant growth-promoting rhizobacteria (PGPR) as a plant growth enhancer for sustainable agriculture: a review. Bacteria 3, 59–75. 10.3390/bacteria3020005

[B56] Hasegawa T. Lucob-Agustin N. Yasufuku K. Kojima T. Nishiuchi S. Ogawa A. (2021). Mutation of OUR1/OsbZIP1, which encodes a member of the basic leucine zipper transcription factor family, promotes root development in rice through repressing auxin signaling. Plant Sci. 306, 110861. 10.1016/j.plantsci.2021.110861 33775366

[B57] He X. Qu B. Li W. Zhao X. Teng W. Ma W. (2015). The nitrate-inducible NAC transcription factor TaNAC2-5A controls nitrate response and increases wheat yield. Plant Physiol. 169, 1991–2005. 10.1104/pp.15.00568 26371233 PMC4634051

[B58] Hirani A. H. Asif M. Sharma M. Basu S. K. Iqbal M. Sajad M. (2014). “Genomics, transcriptomics, and molecular breeding for improving cereals,” in Omics Technologies and Crop Improvement (Boca Raton, FL, USA: CRC Press), 303–322.

[B59] Hirel B. Tétu T. Lea P. J. Dubois F. (2011). Improving nitrogen use efficiency in crops for sustainable agriculture. Sustainability 3, 1452–1485. 10.3390/su3091452

[B60] Hossain M. R. Bassel G. W. Pritchard J. Sharma G. P. Ford-Lloyd B. V. (2016). Trait-specific expression profiling of salt stress-responsive genes in diverse rice genotypes as determined by modified significance analysis of microarrays. Front. Plant Sci. 7, 567. 10.3389/fpls.2016.00567 27200040 PMC4853522

[B61] Hou M. Yu M. Li Z. Ai Z. Chen J. (2021). Molecular regulatory networks for improving nitrogen use efficiency in rice. Int. J. Mol. Sci. 22, 9040. 10.3390/ijms22169040 34445746 PMC8396546

[B62] Htwe NMPS Ruangrak E. (2021). A review of sensing, uptake, and environmental factors influencing nitrate accumulation in crops. J. Plant Nutr. 44 (7), 1054–1065. 10.1080/01904167.2021.1871757

[B63] Hu B. Wang W. Ou S. Tang J. Li H. Che R. (2015). Variation in NRT1.1NRT1.1B contributes to nitrate-use divergence between rice subspecies. Nat. Genet. 47, 834–838. 10.1038/ng.3337 26053497

[B64] Hu Q. Q. Shu J. Q. Li W. M. Wang G. Z. (2021). Role of auxin and nitrate signaling in the development of root system architecture. Front. Plant Sci. 12, 690363. 10.3389/fpls.2021.690363 34858444 PMC8631788

[B65] Huang T. Sun R. Wu Q. Zhang X. Li J. Zhou B. (2025). Assessment of genetic improvements in wheat yield and nitrogen use efficiency under different nitrogen input levels: a global perspective. Eur. J. Agron. 171, 127813. 10.1016/j.eja.2025.127813

[B66] Ibn R. A. Ghosh U. K. Hossain M. S. Mahmud A. Saha A. K. Rahman M. M. (2024). Enhancing nitrogen use efficiency in cereal crops: from agronomy to genomic perspectives. Cereal Res. Commun. 53, 1–16. 10.1007/s42976-024-00515-5

[B67] Ibn R. A. Ghosh U. K. Hossain M. S. Mahmud A. Saha A. K. Rahman M. M. (2025). Enhancing nitrogen use efficiency in cereal crops from agronomy to genomic perspectives. Cereal Res. Commun. 53 (1), 1–16.

[B68] Ibrahim A. A. Abd-Ellatif S. Abdel Razik E. S. S. Salem K. F. (2024). “Biodiversity of cereal crops and utilization in food and nutritional security,” in Sustainable Utilization and Conservation of Plant Genetic Diversity (Singapore: Springer Nature Singapore), 31–61.

[B69] Ishfaq J. Soomar A. M. Khalid F. Abbasi Y. (2023). Assessing rice (Oryza sativa L.) quality: a comprehensive review of current techniques and future directions. J. Agric. Food Res. 14, 100843. 10.1016/j.jafr.2023.100843

[B70] Islam M. Q. Hasan M. N. Hoque H. Jewel N. A. Bhuiyan M. F. H. Prodhan S. H. (2021). Characterization of transcription factor MYB59 and expression profiling in response to low K^+^ and NO_3_ ^-^ in indica rice (Oryza sativa L.) J. Genet. Eng. Biotechnol. 19, 167. 10.1186/s43141-021-00248-6 34704216 PMC8548439

[B71] Islam S. Islam R. Kandwal P. Khanam S. Proshad R. Kormoker T. (2022). Nitrate transport and assimilation in plants: a potential review. Archives Agron. Soil Sci. 68 (1), 133–150. 10.1080/03650340.2020.1826042

[B72] Iwamoto M. Tagiri A. (2016). MicroRNA-targeted transcription factor gene RDD1 promotes nutrient ion uptake and accumulation in rice. Plant J. 85, 466–477. 10.1111/tpj.13117 26729506

[B73] Jagadhesan B. Sathee L. Meena H. S. Jha S. K. Chinnusamy V. Kumar A. (2020). Genome wide analysis of NLP transcription factors reveals their role in nitrogen stress tolerance of rice. Sci. Rep. 10, 9368. 10.1038/s41598-020-66338-6 32523127 PMC7287097

[B74] Jaikumar N. S. Stutz S. S. Fernandes S. B. Leakey A. D. Bernacchi C. J. Brown P. J. (2021). Can improved canopy light transmission ameliorate loss of photosynthetic efficiency in the shade? An investigation of natural variation in Sorghum bicolor. J. Exp. Bot. 72, 4965–4980. 10.1093/jxb/erab176 33914063 PMC8219039

[B75] Jain D. Jones L. Roy S. (2023). Gene editing to improve legume-rhizobia symbiosis in a changing climate. Curr. Opin. Plant Biol. 71, 102324. 10.1016/j.pbi.2022.102324 36535148

[B76] Jan M. F. Li M. Liu C. Liaqat W. Altaf M. T. Barutçular C. (2025). Multivariate analysis of root architecture, morpho-physiological, and biochemical traits reveals higher nitrogen use efficiency heterosis in maize hybrids during early vegetative growth. Plants 14 (3), 399. 10.3390/plants14030399 39942961 PMC11821247

[B77] Jat S. L. Suby S. B. Parihar C. M. Gambhir G. Kumar N. Rakshit S. (2021). Microbiome for sustainable agriculture: a review with special reference to the corn production system. Archives Microbiol. 203, 2771–2793. 10.1007/s00203-021-02320-8 33884458

[B78] Javed T. I I. Singhal R. K. Shabbir R. Shah A. N. Kumar P. (2022). Recent advances in agronomic and physio-molecular approaches for improving nitrogen use efficiency in crop plants. Front. Plant Sci. 13, 877544. 10.3389/fpls.2022.877544 35574130 PMC9106419

[B79] Jia L. Hu D. Wang J. Liang Y. Li F. Wang Y. (2023). Genome-wide identification and functional analysis of nitrate transporter genes (NPF, NRT2, and NRT3) in maize. Int. J. Mol. Sci. 24 (16), 12941. 10.3390/ijms241612941 37629121 PMC10454388

[B80] Jia Y. Xu N. Wu J. Wang C. Huang M. Li Y. (2025). Genome-wide association study, linkage mapping and transcriptomic analysis revealed candidate genes with the flag leaf traits associated with nitrogen use efficiency in wheat. BMC Genomics 26 (1), 833. 10.1186/s12864-025-12025-7 41013191 PMC12466056

[B81] Jiang L. Ball G. Hodgman C. Coules A. Zhao H. Lu C. (2018). Analysis of gene regulatory networks of maize in response to nitrogen. Genes 9, 151. 10.3390/genes9030151 29518046 PMC5867872

[B82] Kabir M. Y. Nambeesan S. U. Díaz-Pérez J. C. (2024). Shade nets improve vegetable performance. Sci. Hortic. 334, 113326. 10.1016/j.scienta.2024.113326

[B83] Kalra A. Goel S. Elias A. A. (2024). Understanding role of roots in plant response to drought: way forward to climate‐resilient crops. Plant Genome 17, e20395. 10.1002/tpg2.20395 37853948 PMC12806938

[B84] Kan C. C. Chung T. Y. Juo Y. A. Hsieh M. H. (2015). Glutamine rapidly induces the expression of key transcription factor genes involved in nitrogen and stress responses in rice roots. BMC Genomics 16, 1–15. 10.1186/s12864-015-1892-7 26407850 PMC4582844

[B85] Karunarathne S. D. Han Y. Zhang X. Q. Li C. (2022). CRISPR/Cas9 gene editing and natural variation analysis demonstrate the potential for HvARE1ARE1 in improvement of nitrogen use efficiency in barley. J. Integr. Plant Biol. 64, 756–770. 10.1111/jipb.13214 35014191

[B86] Katiyar P. Kumar S. Arora N. K. (2022). “Interactions of nitrogen-fixing bacteria and cereal crops: an important dimension,” in Nitrogen Fixing Bacteria: Sustainable Growth of Non-legumes (Singapore: Springer Nature), 169–194.

[B87] Kaur M. Tak Y. Bhatia S. Asthir B. Lorenzo J. M. Amarowicz R. (2021). Crosstalk during the carbon–nitrogen cycle that interlinks the biosynthesis, mobilization, and accumulation of seed storage reserves. Int. J. Mol. Sci. 22 (21), 12032. 10.3390/ijms222112032 34769462 PMC8585027

[B88] Khan Y. Shah S. Tian H. (2022). The roles of arbuscular mycorrhizal fungi in influencing plant nutrients, photosynthesis, and metabolites of cereal crops—A review. Agronomy 12 (9), 2191. 10.3390/agronomy12092191

[B89] Khan A. A. Iqbal B. Jalal A. Khan K. A. Al-Andal A. Khan I. (2024). Advanced molecular approaches for improving crop yield and quality: a review. J. Plant Growth Regul. 43, 2091–2103. 10.1007/s00344-024-11050-x

[B90] Khong G. N. Richaud F. Coudert Y. Pati P. K. Santi C. Périn C. (2008). Modulating rice stress tolerance by transcription factors. Biotechnol. Genet. Eng. Rev. 25, 381–404. 10.1080/02648720802637003 21412363

[B91] Kong L. Zhang Y. Du W. Xia H. Fan S. Zhang B. (2021). Signaling responses to N starvation: focusing on wheat and filling the putative gaps with findings obtained in other plants. A review. Front. Plant Sci. 12, 656696. 10.3389/fpls.2021.656696 34135921 PMC8200679

[B92] Konishi M. Yanagisawa S. (2013). Arabidopsis NIN-like transcription factors have a central role in nitrate signalling. Nat. Commun. 4, 1617. 10.1038/ncomms2647 23511481

[B93] Kumar R. (2014). Role of microRNAs in biotic and abiotic stress responses in crop plants. Appl. Biochem. Biotechnol. 174, 93–115. 10.1007/s12010-014-0841-3 24869742

[B94] Kumar A. Batra R. Gahlaut V. Gautam T. Kumar S. Sharma M. (2018). Genome-wide identification and characterization of gene family for RWP-RK transcription factors in wheat (*Triticum aestivum* L.). PLoS ONE 13, e0208409. 10.1371/journal.pone.0208409 30540790 PMC6291158

[B95] Kumar A. Naqvi S. D. Y. Kaushik P. Khojah E. Amir M. Alam P. (2022). Rhizophagus irregularis and nitrogen-fixing Azotobacter enhance greater yam (Dioscorea alata) biochemical profile and uphold yield under reduced fertilization. Saudi J. Biol. Sci. 29 (5), 3694–3703. 10.1016/j.sjbs.2022.03.001 35844423 PMC9280223

[B96] Kumar V. Rithesh L. Raghuvanshi N. Kumar A. Parmar K. (2024). Advancing nitrogen use efficiency in cereal crops: a comprehensive exploration of genetic manipulation, nitrogen dynamics, and plant nitrogen assimilation. S. Afr. J. Bot. 169, 486–498. 10.1016/j.sajb.2024.02.003

[B97] Kumar Singh A. Singh R. Subramani R. Kumar R. Wankhede D. P. (2016). Molecular approaches to understand nutritional potential of coarse cereals. Curr. Genom. 17, 177–192. 10.2174/1389202917666160104120051 PMC4869005 27252585

[B98] Kumari S. Sharma N. Raghuram N. (2021). Meta-analysis of yield-related and N-responsive genes reveals chromosomal hotspots, key processes and candidate genes for nitrogen-use efficiency in rice. Front. Plant Sci. 12, 627955. 10.3389/fpls.2021.627955 34168661 PMC8217879

[B99] Kumari R. Bhatnagar S. Kalra C. (2022). “Nitrogen assimilation in plants,” in Advances in Plant Nitrogen Metabolism (Boca Raton, FL: CRC Press), 38–54.

[B100] Kunwar U. B. Manzoor N. Wen J. Pandit N. R. (2025). Integrating agronomic and molecular advancements to enhance nitrogen use efficiency (NUE) and promote sustainable rice production. Nitrogen 6 (2), 34. 10.3390/nitrogen6020034

[B101] Kurai T. Wakayama M. Abiko T. Yanagisawa S. Aoki N. Ohsugi R. (2011). Introduction of the ZmDof1 gene into rice enhances carbon and nitrogen assimilation under low-nitrogen conditions. Plant Biotechnol. J. 9, 826–837. 10.1111/j.1467-7652.2011.00618.x 21624033

[B102] Ladha J. K. Pathak H. Krupnik T. J. Six J. van Kessel C. (2005). Efficiency of fertilizer nitrogen in cereal production: retrospects and prospects. Adv. Agron. 87, 85–156. 10.1016/S0065-2113(05)87003-0

[B103] Lal S. K. Gaggar P. Kumar S. Mallikarjuna M. G. Vishwakarma C. Rakshit S. (2024). Recent advancements in nitrogen use efficiency in crop plants achieved by genomics and targeted genetic engineering approaches. Plant Mol. Biol. Rep. 42, 435–449. 10.1007/s11105-024-01472-1

[B104] Lata J. C. Le Roux X. Koffi K. F. Yé L. Srikanthasamy T. Konaré S. (2022). The causes of the selection of biological nitrification inhibition (BNI) in relation to ecosystem functioning and a research agenda to explore them. Biol. Fertil. Soils 58, 207–224. 10.1007/s00374-021-01608-x

[B105] Lawrenson T. Shorinola O. Stacey N. Li C. Østergaard L. Patron N. (2015). Induction of targeted, heritable mutations in barley and *Brassica oleracea* using RNA-guided Cas9 nuclease. Genome Biol. 16, 258. 10.1186/s13059-015-0816-5 26616834 PMC4663725

[B106] Lebedev V. G. Popova A. A. Shestibratov K. A. (2021). Genetic engineering and genome editing for improving nitrogen use efficiency in plants. Cells 10, 3303. 10.3390/cells10123303 34943810 PMC8699818

[B107] Li Q. Gao L. Liu D. Xu L. Zhang X. Zhang C. (2021). Novel insights of maize structural feature in China. Euphytica 217, 7. 10.1007/s10681-020-02750-1

[B108] Li B. Zhang X. Morita S. Sekiya N. Araki H. Gu H. (2022). Are crop deep roots always beneficial for combating drought: a review of root structure and function, regulation and phenotyping. Agric. Water Manag. 271, 107781. 10.1016/j.agwat.2022.107781

[B109] Li H. Zhu X. Wang J. Wei Y. Nai F. Yu H. (2024). Unraveling differential characteristics and mechanisms of nitrogen uptake in wheat cultivars with varied nitrogen use efficiency. Plant Physiology Biochem. 206, 108278. 10.1016/j.plaphy.2023.108278 38147707

[B110] Liao H. S. Chung Y. H. Hsieh M. H. (2022). Glutamate: a multifunctional amino acid in plants. Plant Sci. 318, 111238. 10.1016/j.plantsci.2022.111238 35351313

[B111] Liu L. White M. J. MacRae T. H. (1999). Transcription factors and their genes in higher plants: functional domains, evolution and regulation. Eur. J. Biochem. 262, 247–257. 10.1046/j.1432-1327.1999.00249.x 10336605

[B112] Liu Z. Zhu C. Jiang Y. Tian Y. Yu J. An H. (2016). Association mapping and genetic dissection of nitrogen use efficiency-related traits in rice (Oryza sativa L.). Funct. Integr. Genom. 16, 323–333. 10.1007/s10142-016-0498-2 26922174

[B113] Liu Q. Wu K. Song W. Zhong N. Wu Y. Fu X. (2022). Improving crop nitrogen use efficiency toward sustainable green revolution. Annu. Rev. Plant Biol. 73, 523–551. 10.1146/annurev-arplant-102421-084643 35595292

[B114] Liu X. Jiang H. Yang J. Han J. Jin M. Zhang H. (2022). Comprehensive QTL analyses of nitrogen use efficiency in indica rice. Front. Plant Sci. 13, 992225. 10.3389/fpls.2022.992225 36212385 PMC9539535

[B115] Liu Y. Hu B. Chu C. (2023). Toward improving nitrogen use efficiency in rice: utilization, coordination, and availability. Curr. Opin. Plant Biol. 71, 102327. 10.1016/j.pbi.2023.102327 36525788

[B116] Liu Q. Zhang Z. Bai C. Yin X. Lin W. Yao L. (2024). Inhibition of microelement accumulation and disorder of saccharide and amino acid metabolism explain rice grain empty under dimethylarsinic acid stress. Plant Cell Rep. 43 (8), 199. 10.1007/s00299-024-03244-w 39039362

[B117] Liu H. Gao X. Fan W. Fu X. (2025). Optimizing carbon and nitrogen metabolism in plants: from fundamental principles to practical applications. J. Integr. Plant Biol. 67 (6), 1447–1466. 10.1111/jipb.13919 40376749

[B118] Liu Z. Wang Z. Wu G. Chen J. He J. Wu M. (2025). Optimizing the ratio of one-off slow-release fertilizer can improve the nitrogen use efficiency and yield of rice under the condition of nitrogen reduction. Plants 14 (23), 3650. 10.3390/plants14233650 41375359 PMC12694212

[B119] Ludemann C. I. Hijbeek R. van Loon M. P. Murrell T. S. Dobermann A. van Ittersum M. K. (2022). Estimating maize harvest index and nitrogen concentrations in grain and residue using globally available data. Field Crops Res. 284, 108578. 10.1016/j.fcr.2022.108578

[B120] Lyu H. Li Y. Wang Y. Wang P. Shang Y. Yang X. (2024). Drive soil nitrogen transformation and improve crop nitrogen absorption and utilization—A review of green manure applications. Front. Plant Sci. 14, 1305600. 10.3389/fpls.2024.1305600 38239220 PMC10794358

[B121] Ma Z. Wang W. (2025). Identification of key transcription factors involved in root architecture of barley. Triticeae Genomics Genet. 16, 175–183. 10.5376/tgg.2025.16.0019

[B122] Ma J. Zhang K. Fang B. Wang X. Wang S. Jiang L. (2025). Optimization of nitrogen allocation and remobilization improves nitrogen use efficiency of winter wheat in the North China plain. Eur. J. Agron. 171, 127782. 10.1016/j.eja.2025.127782

[B123] Mahboob W. Yang G. Irfan M. (2023). Crop nitrogen (N) utilization mechanism and strategies to improve N use efficiency. Acta Physiol. Plant. 45 (4), 52. 10.1007/s11738-023-03527-6

[B124] Mallikarjuna B. P. Shettigar N. Radhika D. H. Devi E. L. Bhat J. S. Patil B. S. (2022). “Genome-wide association studies and genomic selection for nutrient use efficiency in cereals,” in Next-Generation Plant Breeding Approaches for Stress Resilience in Cereal Crops (Singapore: Springer Nature), 161–197. 10.1007/978-981-19-0328-0_9

[B125] Maqbool S. Hassan M. A. Xia X. York L. M. Rasheed A. He Z. (2022). Root system architecture in cereals: progress, challenges and perspective. Plant J. 110, 23–42. 10.1111/tpj.15610 35020968

[B126] Mazahar S. Umar S. (2025). “Dynamic function of miRNAs in sensing and signaling nutrient stress in plants,” in Mirnaomics and Stress Management in Plants (Boca Raton, FL: CRC Press), 55–64.

[B127] McKevith B. (2004). Nutritional aspects of cereals. Nutr. Bull. 29, 111–142. 10.1111/j.1467-3010.2004.00435.x

[B128] Mehta D. Vyas S. Dudhagara D. Patel A. Parmar V. (2024). Significance of Indian millets in enhancing global food security: a comprehensive review. Trends Food Sci. Technol. 149, 104527. 10.1016/j.tifs.2024.104527

[B129] Mihrete T. B. Mihretu F. B. (2025). Crop diversification for ensuring sustainable agriculture, risk management, and food security. Glob. Challenges 2400267 9, 2400267. 10.1002/gch2.202400267 PMC11802337 39925666

[B130] Mishra S. Singroha G. Tiwari R. Sharma P. (2025). Comprehensive genome-wide expression analysis of NLP transcription factors elucidates their crucial role in enhancing nitrogen response in wheat (*Triticum aestivum* L.). Curr. Plant Biol. 42, 100463. 10.1016/j.cpb.2025.100463

[B131] Mohammed N. A. A. (2018). Exploring Rice Genetic Resources to Improve Nutrient Use Efficiency. York, UK: University of York. Ph.D. Thesis.

[B132] Mohapatra P. K. Sarkar R. K. Panda D. Kariali E. (2025). “Tiller development in rice,” in Tillering Behavior of Rice Plant (Singapore: Springer Nature), 141–159.

[B133] Mohn M. A. (2019). Plant Nitrate Reductase and its Isoform Specific Roles in Nitrate and Nitrite Reduction. Universität zu Köln. Doctoral Dissertation.

[B134] Moroyoqui Parra M. A. (2022). Identifying Canopy Architecture Traits to Optimize Light and Increase radiation-use Efficiency and Grain Yield in Wheat. Ph.D. Thesis. Nottingham, UK: University of Nottingham.

[B135] Mudiyanselage S. D. K. T. (2021). Genetic Improvement of Nitrogen Use Efficiency in Barley. Murdoch, Australia: Murdoch University. Ph.D. Thesis.

[B136] Muller J. De Rosa D. Friedl J. De Antoni Migliorati M. Rowlings D. Grace P. (2023). Combining nitrification inhibitors with a reduced N rate maintains yield and reduces N2O emissions in sweet corn. Nutrient Cycl. Agroecosyst. 125 (2), 107–121. 10.1007/s10705-021-10185-y

[B137] Muratore C. Espen L. Prinsi B. (2021). Nitrogen uptake in plants: the plasma membrane root transport systems from a physiological and proteomic perspective. Plants 10, 681. 10.3390/plants10040681 33916130 PMC8066207

[B138] Murchie E. H. Burgess A. J. (2022). Casting light on the architecture of crop yield. Crop Environ. 1, 74–85. 10.1016/j.crope.2022.03.009

[B139] Murchie E. H. Chen Y. Z. Hubbart S. Peng S. Horton P. (1999). Interactions between senescence and leaf orientation determine *in situ* patterns of photosynthesis and photoinhibition in field-grown rice. Plant Physiol. 119, 553–564. 10.1104/pp.119.2.553 9952451 PMC32132

[B140] Murchie E. H. Reynolds M. Slafer G. A. Foulkes M. J. Acevedo-Siaca L. McAusland L. (2023). A ‘wiring diagram’ for source strength traits impacting wheat yield potential. J. Exp. Bot. 74, 72–90. 10.1093/jxb/erad429 36264277 PMC9786870

[B141] Mytton L. R. Skøt L. (1993). “Breeding for improved symbiotic nitrogen fixation,” in Plant Breeding: Principles and Prospects. Editors Hayward M. D. Bosemark N. O. Romagosa I. Cerezo M. (Netherlands, Dordrecht: Springer), 451–472. 10.1007/978-94-011-1524-7_26

[B142] Nacry P. Bouguyon E. Gojon A. (2013). Nitrogen acquisition by roots: physiological and developmental mechanisms ensuring plant adaptation to a fluctuating resource. Plant Soil 370, 1–29. 10.1007/s11104-013-1645-9

[B143] Nazish T. Arshad M. Jan S. U. Javaid A. Khan M. H. Naeem M. A. (2022). Transporters and transcription factors gene families involved in improving nitrogen use efficiency (NUE) and assimilation in rice (Oryza sativa L.). Transgenic Res. 31 (1), 23–42. 10.1007/s11248-021-00284-5 34524604

[B144] Nedelyaeva O. I. Khramov D. E. Balnokin Y. V. Volkov V. S. (2024). Functional and molecular characterization of plant nitrate transporters belonging to NPF (NRT1/PTR) 6 subfamily. Int. J. Mol. Sci. 25, 13648. 10.3390/ijms252213648 39769409 PMC11677463

[B145] Nguyen T. B. A. Lefoulon C. Nguyen T. H. Blatt M. R. Carroll W. (2023). Engineering stomata for enhanced carbon capture and water-use efficiency. Trends Plant Sci. 28, 1290–1309. 10.1016/j.tplants.2023.08.004 37423785

[B146] Niazi A. Iranbakhsh A. Esmaeel Zadeh M. Ebadi M. Oraghi Ardebili Z. (2023). Zinc oxide nanoparticles (ZnONPs) influenced seed development, grain quality, and remobilization by affecting the transcription of microRNA 171 (miR171), miR156, NAM, and SUT genes in wheat (Triticum aestivum): a biological advantage and risk assessment study. Protoplasma 260, 839–851. 10.1007/s00709-023-01812-4 36318315

[B147] Niinemets Ü. (2010). A review of light interception in plant stands from leaf to canopy in different plant functional types and in species with varying shade tolerance. Ecol. Res. 25, 693–714. 10.1007/s11284-010-0712-3

[B148] Ohri P. Bhardwaj R. Bali S. Kaur R. Jasrotia S. Khajuria A. (2015). The common molecular players in plant hormone crosstalk and signaling. Curr. Protein Pept. Sci. 16, 369–388. 10.2174/1389203716666150207151030 25824391

[B149] Ohyama T. (2010). “Nitrogen as a major essential element of plants,” in Nitrogen Assimilation in Plants, 1–17.

[B150] Olaniyi A. S. (2024). Enhancing Wheat Yield and Protein Formation: Assessing N Uptake Dynamics in Diverse Genotypes. Stillwater, OK, USA: Oklahoma State University. Master’s Thesis.

[B151] Padhan B. K. Sathee L. Kumar S. Chinnusamy V. Kumar A. (2023). Variation in nitrogen partitioning and reproductive stage nitrogen remobilization determines nitrogen grain production efficiency (NUEg) in diverse rice genotypes under varying nitrogen supply. Front. Plant Sci. 14, 1093581. 10.3389/fpls.2023.1093581 36938028 PMC10020356

[B152] Pal N. Nayak J. K. Yadav R. R. Tyagi S. Joshi P. Kumar S. (2023). “Potential applications of CRISPR/Cas9-mediated genome-editing approaches in improvements of cereals,” in Omics and System Biology Approaches for Delivering Better Cereals (Boca Raton, FL, USA: CRC Press), 111–132.

[B153] Parwez R. Aftab T. Gill S. S. Naeem M. (2022). Abscisic acid signaling and crosstalk with phytohormones in regulation of environmental stress responses. Environ. Exp. Bot. 199, 104885. 10.1016/j.envexpbot.2022.104885

[B154] Patel A. Babu S. Rathore S. S. Gairola A. Kumar V. Shekhawat K. (2025). “Crop diversification in India: implications to food security and soil health,” in Agricultural Diversification for Sustainable Food Production (Singapore: Springer Nature), 1–29. 10.1007/978-981-55-6758-0_1

[B155] Phan N. T. H. Draye X. Pham C. V. Bertin P. (2023). Identification of quantitative trait loci controlling nitrogen use efficiency-related traits in rice at the seedling stage under salt condition by genome-wide association study. Front. Plant Sci. 14, 1197271. 10.3389/fpls.2023.1197271 37575915 PMC10415682

[B156] Poutanen K. S. Kårlund A. O. Gómez-Gallego C. Johansson D. P. Scheers N. M. Marklinder I. M. (2022). Grains–a major source of sustainable protein for health. Nutr. Rev. 80, 1648–1663. 10.1111/nure.13195 34741520 PMC9086769

[B157] Prashar A. Compton L. Stromvik M. Tai H. H. (2022). High-Throughput Phenotyping for Crop Improvement and Breeding (Lausanne, Switzerland: Frontiers Media SA).

[B158] Pretty J. (2008). Agricultural sustainability: concepts, principles and evidence. Philos. Trans. R. Soc. B Biol. Sci. 363, 447–465. 10.1098/rstb.2007.2163 17652074 PMC2610163

[B159] Purswani J. Llorente C. P. (2021). “Nitrification and denitrification processes: environmental impacts,” in Nitrogen Cycle (Boca Raton, FL, USA: CRC Press), 60–81.

[B160] Qi J. Yu L. Ding J. Ji C. Wang S. Wang C. (2023). Transcription factor OsSNAC1 positively regulates nitrate transporter gene expression in rice. Plant Physiol. 192, 2923–2942. 10.1093/plphys/kiad420 37204801

[B161] Qin F. Su H. Sun L. Li Y. (2024). Research progress related to sorghum biological nitrification inhibitors. Agronomy 14, 1576. 10.3390/agronomy14081576

[B162] Quemada M. Gabriel J. L. (2016). Approaches for increasing nitrogen and water use efficiency simultaneously. Glob. Food Secur 9, 29–35. 10.1016/j.gfs.2016.06.002

[B163] Rahnama A. Hosseinalipour B. Farrokhian Firouzi A. Tom Harrison M. Ghorbanpour M. (2024). Root architecture traits and genotypic responses of wheat at seedling stage to water-deficit stress. Cereal Res. Commun. 52 (4), 1499–1510. 10.1007/s42976-023-00481-4

[B164] Ranjan A. Sinha R. Singla‐Pareek S. L. Pareek A. Singh A. K. (2022). Shaping the root system architecture in plants for adaptation to drought stress. Physiol. Plantarum 174 (2), e13651. 10.1111/ppl.13651 35174506

[B165] Rastogi M. Kolur S. M. Burud A. Sadineni T. Sekhar M. Kumar R. (2024). Advancing water conservation techniques in agriculture for sustainable resource management: a review. J. Geogr. Environ. Earth Sci. Int. 28, 41–53. 10.9734/jgeesi/2024/v28i3755

[B166] Ravazzolo L. (2019). The maize root response to nitrogen fluctuations: signalling crosstalk with strigolactones. Auxin Transcr. Regul. 01–203.

[B167] Reddy M. B. Sravani P. Kumar S. Rajawat M. V. S. Jaiswal D. K. Dhar S. (2025). Nitrogen use efficiency reimagined: advancements in agronomic, ecophysiological, and molecular strategies. J. Plant Nutr. 48 (9), 1577–1603. 10.1080/01904167.2024.2447840

[B168] Sadhukhan R. Jatav H. S. Sen S. Sharma L. D. Rajput V. D. Thangjam R. (2022). “Biological nitrification inhibition for sustainable crop production,” in Plant Perspectives to Global Climate Changes (Cambridge, MA, USA: Academic Press), 135–150.

[B169] Saengwilai P. Strock C. Rangarajan H. Chimungu J. Salungyu J. Lynch J. P. (2021). Root hair phenotypes influence nitrogen acquisition in maize. Ann. Bot. 128, 849–858. 10.1093/aob/mcab104 34355736 PMC8577201

[B170] Safdar L. B. Foulkes M. J. Kleiner F. H. Searle I. R. Bhosale R. A. Fisk I. D. (2023). Challenges facing sustainable protein production: opportunities for cereals. Plant Commun. 4, 6. 10.1016/j.xplc.2023.100058 PMC10721536 37710958

[B171] Saleem A. Anwar S. Nawaz T. Fahad S. Saud S. Ur Rahman T. (2024). Securing a sustainable future: the climate change threat to agriculture, food security, and sustainable development goals. J. Umm Al-Qura Univ. Appl. Sci. 1, 1–17. 10.1007/s43994-024-00177-3

[B172] Sanchez D. L. Santana A. S. Morais P. I. C. Peterlini E. De La Fuente G. Castellano M. J. (2023). Phenotypic and genome-wide association analyses for nitrogen use efficiency-related traits in maize (Zea mays L.) exotic introgression lines. Front. Plant Sci. 14, 1270166. 10.3389/fpls.2023.1270166 37877090 PMC10590880

[B173] Sandhu N. Sethi M. Kumar A. Dang D. Singh J. Chhuneja P. (2021). Biochemical and genetic approaches improving nitrogen use efficiency in cereal crops: a review. Front. Plant Sci. 12, 657629. 10.3389/fpls.2021.657629 34149755 PMC8213353

[B175] Sathee L. Antoo S. Prabhakar G. D. Arpitha S. R. Kumar S. Watts A. (2023). “CRISPR-based genome editing for improving nutrient use efficiency and functional genomics of nutrient stress adaptation in plants,” in CRISPR and Plant Functional Genomics (Boca Raton, FL: CRC Press), 144–174.

[B176] Sathee L. Nimmy M. S. Singh A. Kumari S. Vinutha T. Durgesh K. (2025). “Epigenomics for nutrient use efficiency: status and future applications for improving crop nitrogen response,” in Non-Coding Rnas for Crop Improvement: Concepts and Applications (Singapore: Springer), 157–173.

[B177] Saud S. Wang D. Fahad S. (2022). Improved nitrogen use efficiency and greenhouse gas emissions in agricultural soils as producers of biological nitrification inhibitors. Front. Plant Sci. 13, 854195. 10.3389/fpls.2022.854195 35432390 PMC9011059

[B178] Shan Q. Wang Y. Li J. Zhang Y. Chen K. Liang Z. (2013). Targeted genome modification of crop plants using a CRISPR-cas system. Nat. Biotechnol. 31, 686–688. 10.1038/nbt.2659 23929338

[B179] Shi H. Chen M. Gao L. Wang Y. Bai Y. Yan H. (2022). Genome-wide association study of agronomic traits related to nitrogen use efficiency in wheat. Theor. Appl. Genet. 135, 4289–4302. 10.1007/s00122-022-04115-3 36136127

[B180] Shilpha J. Song J. Jeong B. R. (2023). Ammonium phytotoxicity and tolerance: an insight into ammonium nutrition to improve crop productivity. Agronomy 13, 1487. 10.3390/agronomy13061487

[B181] Singhal R. K. Jatav H. S. Aftab T. Pandey S. Mishra U. N. Chauhan J. (2021). Roles of nitric oxide in conferring multiple abiotic stress tolerance in plants and crosstalk with other plant growth regulators. J. Plant Growth Regul. 40, 1–26. 10.1007/s00344-021-10365-3

[B182] Sokolowska P. Jost M. Buss W. Ford B. Chandler P. Spielmeyer W. (2025). INDETERMINATE DOMAIN-DELLA protein interactions orchestrate gibberellin-mediated cell elongation in wheat and barley. bioRxiv. 123, 01–10. 10.1101/2025.01.01.123456 PMC12867750 41615756

[B183] Stewart D. W. Costa C. Dwyer L. M. Smith D. L. Hamilton R. I. Ma B. L. (2003). Canopy structure, light interception, and photosynthesis in maize. Agron. J. 95, 1465–1474. 10.2134/agronj2003.1465

[B184] Subbarao G. V. Rondon M. Ito O. Ishikawa T. Rao I. M. Nakahara K. (2007). Biological nitrification inhibition (BNI)—Is it a widespread phenomenon? Plant Soil 294, 5–18. 10.1007/s11104-007-9226-3

[B185] Sun X. Ren W. Wang P. Chen F. Yuan L. Pan Q. (2021). Evaluation of maize root growth and genome-wide association studies of root traits in response to low nitrogen supply at seedling emergence. Crop J. 9, 794–804. 10.1016/j.cj.2021.03.007

[B186] Tang W. Ye J. Yao X. Zhao P. Xuan W. Tian Y. (2019). Genome-wide association study identifies NAC42-activated nitrate transporter conferring high nitrogen use efficiency in rice. Nat. Commun. 10, 5279. 10.1038/s41467-019-13235-0 31754193 PMC6872725

[B187] The S. V. Snyder R. Tegeder M. (2021). Targeting nitrogen metabolism and transport processes to improve plant nitrogen use efficiency. Front. Plant Sci. 11, 628366. 10.3389/fpls.2020.628366 33732269 PMC7957077

[B188] Tiong J. Sharma N. Sampath R. MacKenzie N. Watanabe S. Metot C. (2021). Improving nitrogen use efficiency through overexpression of alanine aminotransferase in rice, wheat, and barley. Front. Plant Sci. 12, 628521. 10.3389/fpls.2021.628521 33584777 PMC7875890

[B189] Tu W. Xu J. Thompson I. P. Huang W. E. (2023). Engineering artificial photosynthesis based on rhodopsin for CO_2_ fixation. Nat. Commun. 14, 8012. 10.1038/s41467-023-44030-6 38049399 PMC10696030

[B190] Tyagi J. Ahmad S. Malik M. (2022). Nitrogenous fertilizers: impact on environmental sustainability, mitigation strategies, and challenges. Int. J. Environ. Sci. Technol. 19, 11649–11672. 10.1007/s13762-022-04312-5

[B191] Vazquez-Carrasquer V. Laperche A. Bissuel-Bélaygue C. Chelle M. Richard-Molard C. (2021). Nitrogen uptake efficiency, mediated by fine root growth, early determines temporal and genotypic variations in nitrogen use efficiency of winter oilseed rape. Front. Plant Sci. 12, 641459. 10.3389/fpls.2021.641459 34054891 PMC8155714

[B192] Verma V. Ravindran P. Kumar P. P. (2016). Plant hormone-mediated regulation of stress responses. BMC Plant Biol. 16, 1–10. 10.1186/s12870-016-0800-5 27079791 PMC4831116

[B193] Vij S. Tyagi A. K. (2007). Emerging trends in the functional genomics of the abiotic stress response in crop plants. Plant Biotechnol. J. 5, 361–380. 10.1111/j.1467-7652.2007.00244.x 17430544

[B194] Vinci G. Ruggieri R. Ruggeri M. Zaki M. G. (2022). Application of life cycle assessment (LCA) to cereal production: an overview. IOP Conf. Ser. Earth Environ. Sci. 1077, 012004. 10.1088/1755-1315/1077/1/012004

[B195] Wang R. F. An D. G. Hu C. S. Li L. H. Zhang Y. M. Jia Y. G. (2011). Relationship between nitrogen uptake and use efficiency of winter wheat grown in the north China plain. Crop and Pasture Sci. 62 (6), 504–514. 10.1071/cp10383

[B196] Wang Q. Nian J. Xie X. Yu H. Zhang J. Bai J. (2018). Genetic variations in ARE1ARE1 mediate grain yield by modulating nitrogen utilization in rice. Nat. Commun. 9, 1–10. 10.1038/s41467-018-03460-7 29467406 PMC5821702

[B197] Wang D. Xu T. Yin Z. Wu W. Geng H. Li L. (2020). Overexpression of OsMYB305 in rice enhances the nitrogen uptake under low-nitrogen condition. Front. Plant Sci. 11, 369. 10.3389/fpls.2020.00369 32351516 PMC7174616

[B198] Wang M. Hasegawa T. Hayashi M. Ohmori Y. Yano K. Teramoto S. (2020). OsNLP4 is required for nitrate assimilation gene expressions and nitrate-dependent growth in rice. bioRxiv, 2020. 10.1101/2020.03.16.993733

[B199] Wang Y. Xu J. Ge M. Ning L. Hu M. Zhao H. (2020). High-resolution profile of transcriptomes reveals a role of alternative splicing for modulating response to nitrogen in maize. BMC Genom 21, 1–19. 10.1186/s12864-020-06819-4 32393171 PMC7216474

[B200] Wang P. Chai Y. N. Roston R. Dayan F. E. Schachtman D. P. (2021). Sorghum bicolor root exudate sorgoleone shapes bacterial communities and delays network formation. mSystems 6, e10–e1128. 10.1128/mSystems.00128-21 PMC8546980 33727394

[B201] Wang W. Li A. Zhang Z. Chu C. (2021). Posttranslational modifications: regulation of nitrogen utilization and signaling. Plant Cell Physiol. 62, 543–552. 10.1093/pcp/pcab198 33493288 PMC8462382

[B202] Wang X. Bai J. Xie T. Wang W. Zhang G. Yin S. (2021). Effects of biological nitrification inhibitors on nitrogen use efficiency and greenhouse gas emissions in agricultural soils: a review. Ecotoxicol. Environ. Saf. 220, 112338. 10.1016/j.ecoenv.2021.112338 34015632

[B203] Wang R. Sun C. Cai S. Liu F. Xie H. Xiong Q. (2023). Research progress in crop root biology and nitrogen uptake and use, with emphasis on cereal crops. Agronomy 13 (7), 1678. 10.3390/agronomy13071678

[B204] Wang M. Wang J. Wang Z. Teng Y. (2024). Nitrate signaling and its role in regulating flowering time in *Arabidopsis thaliana* . Int. J. Mol. Sci. 25, 5310. 10.3390/ijms25105310 38791350 PMC11120727

[B205] Wang Y. Zhang Y. Qiao H. Zheng Y. Hou X. Shi L. (2024). An integrated transcriptome and physiological analysis of nitrogen use efficiency in rice (Oryza sativa L. ssp. Indica) under drought stress. Front. Genet. 15, 1483113. 10.3389/fgene.2024.1483113 39553474 PMC11564168

[B206] Wang J. Chen Y. Shu G. Zhao M. Zheng A. Chang X. (2025). Fast3VmrMLM: a fast algorithm that integrates genome-wide scanning with machine learning to accelerate gene mining and breeding by design for polygenic traits in large-scale GWAS datasets. Plant Commun. 6 (7), 101385. 10.1016/j.xplc.2025.101385 40411144 PMC12281254

[B207] Wasaya A. Zhang X. Fang Q. Yan Z. (2018). Root phenotyping for drought tolerance: a review. Agronomy 8 (11), 241. 10.3390/agronomy8110241

[B208] Wei D. Cui K. Ye G. Pan J. Xiang J. Huang J. (2012). QTL mapping for nitrogen-use efficiency and nitrogen-deficiency tolerance traits in rice. Plant Soil 359, 281–295. 10.1007/s11104-012-1077-0

[B209] Whetton R. L. Harty M. A. Holden N. M. (2022). Communicating nitrogen loss mechanisms for improving nitrogen use efficiency management, focused on global wheat. Nitrogen 3, 213–246. 10.3390/nitrogen3020013

[B210] Wu J. Zhang Z. S. Xia J. Q. Alfatih A. Song Y. Huang Y. J. (2021). Rice NIN‐LIKE PROTEIN 4 plays a pivotal role in nitrogen use efficiency. Plant Biotechnol. J. 19, 448–461. 10.1111/pbi.13455 32876985 PMC7955889

[B211] Wu Q. Xu J. Zhao Y. Wang Y. Zhou L. Ning L. (2024). Transcription factor ZmEREB97 regulates nitrate uptake in maize (Zea mays) roots. Plant Physiol. 196, 535–550. 10.1093/plphys/kad898 38743701 PMC11376383

[B213] Xiong Q. Hu J. Wei H. Zhang H. Zhu J. (2021). Relationship between plant roots, rhizosphere microorganisms, and nitrogen with special focus on rice. Agriculture 11, 234. 10.3390/agriculture11030234

[B214] Xue J. Gou L. Zhao Y. Yao M. Yao H. Tian J. (2016). Effects of light intensity within the canopy on maize lodging. Field Crops Res. 188, 133–141. 10.1016/j.fcr.2016.03.014

[B215] Yadav M. R. Kumar S. Lal M. K. Kumar D. Kumar R. Yadav R. K. (2023). Mechanistic understanding of leakage and consequences and recent technological advances in improving nitrogen use efficiency in cereals. Agronomy 13, 527. 10.3390/agronomy13030527

[B241] Yali W. Mitiku T. (2024). Gene pool, classification and its importance in modern crop improvement program. Int. J. Agric. Sci. Food Technol. 10 (2), 068–073.

[B217] Yang X. Xia X. Zhang Z. Nong B. Zeng Y. Xiong F. (2017). QTL mapping by whole genome re-sequencing and analysis of candidate genes for nitrogen use efficiency in rice. Front. Plant Sci. 8, 1634. 10.3389/fpls.2017.01634 28983307 PMC5613164

[B218] Yang X. Li R. Jablonski A. Stovall A. Kim J. Yi K. (2023). Leaf angle as a leaf and canopy trait: rejuvenating its role in ecology with new technology. Ecol. Lett. 26, 1005–1020. 10.1111/ele.14107 37078440

[B219] Yu C. Chen Y. Cao Y. Chen H. Wang J. Bi Y. M. (2018). Overexpression of miR169o, an overlapping microRNA in response to both nitrogen limitation and bacterial infection, promotes nitrogen use efficiency and susceptibility to bacterial blight in rice. Plant Cell Physiol. 59, 1234–1247. 10.1093/pcp/pcy059 29566243

[B220] Yuan S. Li Z. Li D. Yuan N. Hu Q. Luo H. (2015). Constitutive expression of rice microRNA528 alters plant development and enhances tolerance to salinity stress and nitrogen starvation in creeping bentgrass. Plant Physiol. 169, 576–593. 10.1104/pp.15.00727 26224802 PMC4577425

[B221] Zahedi S. M. Karimi M. Venditti A. Zahra N. Siddique K. H. Farooq M. (2025). Plant adaptation to drought stress: the role of anatomical and morphological characteristics in maintaining the water status. J. Soil Sci. Plant Nutr. 25 (1), 409–427. 10.1007/s42729-024-02141-w

[B222] Zayed O. Hewedy O. A. Abdelmoteleb A. Ali M. Youssef M. S. Roumia A. F. (2023). Nitrogen journey in plants: from uptake to metabolism, stress response, and microbe interaction. Biomolecules 13, 1443. 10.3390/biom13101443 37892125 PMC10605003

[B223] Zhang Y. Tan L. Zhu Z. Yuan L. Xie D. Sun C. (2015). TOND1 confers tolerance to nitrogen deficiency in rice. Plant J. 81, 367–376. 10.1111/tpj.12748 25439309 PMC4329406

[B224] Zhang M. Gao M. Zheng H. Yuan Y. Zhou X. Guo Y. (2019). QTL mapping for nitrogen use efficiency and agronomic traits at the seedling and maturity stages in wheat. Mol. Breed. 39, 1–17. 10.1007/s11032-019-0990-4

[B225] Zhang J. Zhang H. Li S. Li J. Yan L. Xia L. (2021). Increasing yield potential through manipulation of an ARE1ARE1 ortholog related to nitrogen use efficiency in wheat by CRISPR/Cas9. J. Integr. Plant Biol. 63, 1649–1663. 10.1111/jipb.13099 34270164

[B226] Zhang Y. Li B. Liu F. Luo P. Wang Y. Liu D. (2021). Transcriptomic and physiological analysis revealed the ammonium tolerance mechanisms of Myriophyllum aquaticum. Environ. Exp. Bot. 187, 104462. 10.1016/j.envexpbot.2021.104462

[B227] Zhang B. Chen L. Jin S. Guo Q. Hou J. (2022). The influence of plants on the migration and transformation of nitrogen in plant-soil systems: a review. J. Soil Sci. Plant Nutr. 22 (4), 4084–4102. 10.1007/s42729-022-01009-1

[B228] Zhang Z. S. Xia J. Q. Alfatih A. Song Y. Huang Y. J. Sun L. Q. (2022). Rice NIN‐LIKE PROTEIN 3 modulates nitrogen use efficiency and grain yield under nitrate‐sufficient conditions. Plant Cell Environ. 45, 1520–1536. 10.1111/pce.14390 35150141

[B229] Zhang H. Jin Z. Cui F. Zhao L. Zhang X. Chen J. (2023). Epigenetic modifications regulate cultivar-specific root development and metabolic adaptation to nitrogen availability in wheat. Nat. Commun. 14, 8238. 10.1038/s41467-023-41215-1 38086830 PMC10716289

[B230] Zhang P. Wang Y. Sheng D. Zhang S. Gu S. Yan Y. (2023). Optimizing root system architecture to improve root anchorage strength and nitrogen absorption capacity under high plant density in maize. Field Crops Res. 303, 109109. 10.1016/j.fcr.2023.109109

[B231] Zhang Y. He Z. Qi X. Li M. Liu J. Le S. (2023). Overexpression of MYB-like transcription factor SiMYB30 from foxtail millet (Setaria italica L.) confers tolerance to low nitrogen stress in transgenic rice. Plant Physiology Biochem. 196, 731–738. 10.1016/j.plaphy.2023.02.025 36822026

[B232] Zhang S. Ji Z. Jiao W. Shen C. Qin Y. Huang Y. (2025). Natural variation of OsWRKY23 drives difference in nitrate use efficiency between indica and japonica rice. Nat. Commun. 16, 1420. 10.1038/s41467-025-01743-x 39915505 PMC11802876

[B233] Zhao Y. Liu J. Li Y. Wen H. Teotia S. Zhang X. (2022). osa-miR528 promotes seedling growth by enhancing nitrate uptake under nitrogen deficiency in rice. Environ. Exp. Bot. 202, 105040. 10.1016/j.envexpbot.2022.105040

[B234] Zhao Y. Islam S. Alhabbar Z. Zhang J. O’Hara G. Anwar M. (2023). Current progress and future prospect of wheat genetics research towards an enhanced nitrogen use efficiency. Plants 12, 1753. 10.3390/plants12101753 37176811 PMC10180859

[B235] Zhou Y. Tao Y. Tang D. Wang J. Zhong J. Wang Y. (2017). Identification of QTL associated with nitrogen uptake and nitrogen use efficiency using high-throughput genotyped CSSLs in rice (Oryza sativa L.). Front. Plant Sci. 8, 1166. 10.3389/fpls.2017.01166 28744289 PMC5504168

[B236] Zhu J. Song N. Sun S. Yang W. Zhao H. Song W. (2016). Efficiency and inheritance of targeted mutagenesis in maize using CRISPR-Cas9. J. Genet. Genom. 43, 25–36. 10.1016/j.jgg.2015.11.004 26842991

[B237] Zhu H. Zhang T. Zhang C. He X. Shi A. Tan W. (2022). Optimizing irrigation and nitrogen management to increase yield and nitrogen recovery efficiency in double-cropping rice. Agronomy 12 (5), 1190. 10.3390/agronomy12051190

[B238] Zhu Q. Zhou G. W. Zhang S. M. (2025). Influence of nitrogen application rates on nitrogen acquisition and utilization efficiency of maize varieties under drip irrigation systems. Appl. Ecol. and Environ. Res. 23 (2), 2377–2391. 10.15666/aeer/2302_23772391

[B239] Zou H. Li D. Ren K. Liu L. Zhang W. Duan Y. (2024). Response of maize yield and nitrogen recovery efficiency to nitrogen fertilizer application in field with various soil fertility. Front. Plant Sci. 15, 1349180. 10.3389/fpls.2024.1349180 38481406 PMC10935998

[B240] Zuluaga D. L. Sonnante G. (2019). The use of nitrogen and its regulation in cereals: structural genes, transcription factors, and the role of miRNAs. Plants 8, 294. 10.3390/plants8110294 31434274 PMC6724420

