# Supplementary material for: Enhancing cereal productivity via nitrogen use efficiency: from conventional breeding to modern genomics
Source: Front Genet. 2026 Jun 22;17:1822936. doi: 10.3389/fgene.2026.1822936 (PMC13333178; doi:10.3389/fgene.2026.1822936)
Supplement: Supplementary file 1 [file Table1.docx]

Supplementary Table 1. QTLs identified in rice, wheat and maize for traits associated with NUE

| **S. No.** | **Crops** | **Traits** | **QTLs Name** | **Markers Name** | **Chromosome** | **Population** | **References** |
| --- | --- | --- | --- | --- | --- | --- | --- |
| 1 | Rice | NCG | *ncg8.1* | 113 SSR and 103 STS | 8 | 166 RILs | Cho *et al*. 2007 |
| 2 |  |  | ncg9 |  | 9 |  |  |
| 3 |  |  | ncg10 |  | 10 |  |  |
| 4 |  |  | ncg8.2 |  | 8 |  |  |
| 5 |  | NCS | ncs9 |  | 9 |  |  |
| 6 |  |  | ncs10 |  | 10 |  |  |
| 7 |  | TNCS | Tncslb |  | 1b |  |  |
| 8 |  |  | tncslO |  | 10 |  |  |
| 9 |  | HI | hi5b |  | 5b |  |  |
| 10 |  |  | hi7b |  | 7b |  |  |
| 11 |  | GY | gy2b |  | 2b |  |  |
| 12 |  |  | gy9 |  | 9 |  |  |
| 13 |  | SY | Sylb |  | 1b |  |  |
| 14 |  |  | sy8.1 |  | 8 |  |  |
| 15 |  |  | sy8.2 |  | 8 |  |  |
| 16 |  |  | sy5a |  | 5a |  |  |
| 17 |  |  | sy5b |  | 5b |  |  |
| 18 |  |  | sy7b |  | 7b |  |  |
| 19 |  |  | syl2b |  | 12b |  |  |
| 20 |  | GYR | qGR3 | 168 RFLP and 52 SSR markers | 3 | 127 RILs | Wei *et al*. 2012 |
| 21 |  |  | qGR9 |  | 9 |  |  |
| 22 |  |  | qGR1-1 |  | 1 |  |  |
| 23 |  |  | qGR1-2 |  | 2 |  |  |
| 24 |  |  | qGR2 |  | 2 |  |  |
| 25 |  | NUE | qNUE2.1 | SNPs | 2 | 128 CSSLs | Zhou *et al*. 2017 |
| 26 |  |  | qNUE4.1 |  | 4 |  |  |
| 27 |  |  | qNUE6.1 |  | 6 |  |  |
| 28 |  |  | qNUE6.2 |  | 6 |  |  |
| 29 |  |  | qNUE10.1 |  | 10 |  |  |
| 30 |  |  | qNUE10.2 |  | 10 |  |  |
| 31 |  | GY | qGY6.1 |  | 6 |  |  |
| 32 |  |  | qGY8.1 |  | 1 |  |  |
| 33 |  | BY | qBY1.1 |  | 1 |  |  |
| 34 |  |  | qBY2.1 |  | 2 |  |  |
| 35 |  |  | qBY2.2 |  | 2 |  |  |
| 36 |  |  | qBY3.1 |  | 3 |  |  |
| 37 |  |  | qBY6.1 |  | 6 |  |  |
| 38 |  |  | qBY8.1 |  | 8 |  |  |
| 39 |  |  | qBY10.1 |  | 10 |  |  |
| 40 |  |  | qBY11.1 |  | 11 |  |  |
| 41 |  | NUE | qNUE6 | 24 simple  sequence repeat | 6 | 280 F2:3 | Yang *et al*. 2017 |
| 42 |  | EPN | qEPN-LN1 | 2,345 Bin marker | 1 | 261 RILs | Liu *et al*. 2022 |
| 43 |  |  | qEPN-LN2 |  | 1 |  |  |
| 44 |  |  | qEPN-LN3 |  | 2 |  |  |
| 45 |  |  | qEPN-LN4 |  | 3 |  |  |
| 46 |  |  | qEPN-LN5 |  | 4 |  |  |
| 47 |  | GNPP | qGNPP-LN1 |  | 1 |  |  |
| 48 |  |  | qGNPPLN2 |  | 2 |  |  |
| 49 |  |  | qGNPPLN3 |  | 3 |  |  |
| 50 |  |  | qGNPPLN4 |  | 4 |  |  |
| 51 |  |  | qGNPPLN5 |  | 5 |  |  |
| 52 |  |  | qGNPPLN6 |  | 7 |  |  |
| 53 |  |  | qGNPPLN7 |  | 12 |  |  |
| 54 |  | TGW | qTGW-LN1 |  | 1 |  |  |
| 55 |  |  | qTGW-LN2 |  | 1 |  |  |
| 56 |  |  | qTGW-LN3 |  | 2 |  |  |
| 57 |  |  | qTGW-LN4 |  | 3 |  |  |
| 58 |  |  | qTGW-LN5 |  | 5 |  |  |
| 59 |  |  | qTGW-LN6 |  | 10 |  |  |
| 60 |  | SSP | qSSP-LN1 |  | 9 |  |  |
| 61 |  | PH | qPH-LN1 |  | 1 |  |  |
| 62 |  |  | qPH-LN2 |  | 5 |  |  |
| 63 |  |  | qPH-LN3 |  | 6 |  |  |
| 64 |  |  | qPH-LN4 |  | 6 |  |  |
| 65 |  |  | qPH-LN5 |  | 7 |  |  |
| 66 |  |  | qPH-LN6 |  | 8 |  |  |
| 67 |  |  | qPH-LN7 |  | 8 |  |  |
| 68 |  |  | qPH-LN8 |  | 10 |  |  |
| 69 |  | PL | qPL-LN1 |  | 1 |  |  |
| 70 |  |  | qPL-LN2 |  | 2 |  |  |
| 71 |  |  | qPL-LN3 |  | 2 |  |  |
| 72 |  |  | qPL-LN4 |  | 3 |  |  |
| 73 |  |  | qPL-LN5 |  | 4 |  |  |
| 74 |  |  | qPL-LN6 |  | 6 |  |  |
| 75 |  | PHR | RM5639 | 157 genome-wide simple sequence repeat (SSR) | 3 | 184 rice accessions | Liu *et al*. 2016 |
| 76 |  |  | RM5748 |  | 3 |  |  |
| 77 |  |  | RM314 |  | 6 |  |  |
| 78 |  |  | RM3628 |  | 6 |  |  |
| 79 |  | TNR | W2 |  | 9 |  |  |
| 80 |  |  | RM519 |  | 12 |  |  |
| 81 |  | GLR | RM518 |  | 4 |  |  |
| 82 |  |  | RM3419 |  | 5 |  |  |
| 83 | Wheat | MRL | QMrl-2B.1 | SNPs | 2B | 188 RIL | Fan *et al*. 2018 |
| 84 |  |  | QMrl-2B.2 |  | 2B |  |  |
| 85 |  |  | QMrl-2D |  | 2D |  |  |
| 86 |  |  | QMrl-3D |  | 2D |  |  |
| 87 |  |  | QMrl-6D.1 |  | 6D |  |  |
| 88 |  |  | QMrl-6D.2 |  | 6D |  |  |
| 89 |  | RDW | QRdw-1D |  | 1D |  |  |
| 90 |  |  | QRdw-2D |  | 2D |  |  |
| 91 |  |  | QRdw-3B |  | 3B |  |  |
| 92 |  |  | QRdw-4A |  | 4A |  |  |
| 93 |  |  | QRdw-4B |  | 4B |  |  |
| 94 |  |  | QRdw-6B |  | 6B |  |  |
| 95 |  |  | QRdw-7B |  | 7B |  |  |
| 96 |  | RL | QRl-1B |  | 1B |  |  |
| 97 |  |  | QRl-1D |  | 1D |  |  |
| 98 |  |  | QRl-2D.1 |  | 2D |  |  |
| 99 |  |  | QRl-2D.2 |  | 2D |  |  |
| 100 |  |  | QRl-3A |  | 3A |  |  |
| 101 |  |  | QRl-6B.1 |  | 6B |  |  |
| 102 |  |  | QRl-6B.2 |  | 6B |  |  |
| 103 |  |  | QRl-7B |  | 7B |  |  |
| 104 |  | RS | QRs-2D |  | 2D |  |  |
| 105 |  |  | QRs-3D |  | 3D |  |  |
| 106 |  |  | QRs-6B |  | 6B |  |  |
| 107 |  | RV | QRv-2D.1 |  | 2D |  |  |
| 108 |  |  | QRv-2D.2 |  | 2D |  |  |
| 109 |  |  | QRv-3A |  | 3A |  |  |
| 110 |  |  | QRv-3D |  | 3D |  |  |
| 111 |  |  | QRv-6B |  | 6B |  |  |
| 112 |  |  | QRv-7A |  | 7A |  |  |
| 113 |  | RD | QRd-1B |  | 1B |  |  |
| 114 |  |  | QRd-2A |  | 2A |  |  |
| 115 |  |  | QRd-2B |  | 2B |  |  |
| 116 |  |  | QRd-3A.1 |  | 3A |  |  |
| 117 |  |  | QRd-3A.2 |  | 3A |  |  |
| 118 |  |  | QRd-3B |  | 3B |  |  |
| 119 |  |  | QRd-3D |  | 3D |  |  |
| 120 |  |  | QRd-5B.1 |  | 5B |  |  |
| 121 |  |  | QRd-5B.2 |  | 5B |  |  |
| 122 |  |  | QRd-6A |  | 6A |  |  |
| 123 |  |  | QRd-6B |  | 6B |  |  |
| 124 |  |  | QRd-6D.1 |  | 6D |  |  |
| 125 |  |  | QRd-6D.2 |  | 6D |  |  |
| 126 |  |  | QRd-7A |  | 7A |  |  |
| 127 |  | RT | QRt-2B |  | 2B |  |  |
| 128 |  |  | QRt-3A.1 |  | 3A |  |  |
| 129 |  |  | QRt-3A.2 |  | 3A |  |  |
| 130 |  |  | QRt-3B |  | 3B |  |  |
| 131 |  |  | QRt-5A |  | 5A |  |  |
| 132 |  |  | QRt-6B |  | 6B |  |  |
| 133 |  |  | QRt-7A |  | 7A |  |  |
| 134 |  |  | QRt-7B |  | 7B |  |  |
| 135 |  | SH | QSh-4B.1 | 1506 SNP and 105 SSR | 4B | 184 RILs | Zhang *et al*. 2019 |
| 136 |  |  | QSh-6B.2 |  | 6B |  |  |
| 137 |  | MRL | QMrl-4A.2 |  | 4A |  |  |
| 138 |  | SDW | QSdw-4B.3 |  | 4B |  |  |
| 139 |  |  | QSdw-4B.4 |  | 4B |  |  |
| 140 |  | TDW | QTdw-4B.2 |  | 4B |  |  |
| 141 |  |  | QTdw-4B.3 |  | 4B |  |  |
| 142 |  |  | QTdw-4B.4 |  | 4B |  |  |
| 143 |  | RSDW | QRsdw-4B.2 |  | 4B |  |  |
| 144 |  |  | QRsdw-4B.3 |  | 4B |  |  |
| 145 |  | SNC | QSnc-4B.2 |  | 4B |  |  |
| 146 |  | TNC | QTnc-4B |  | 4B |  |  |
| 147 |  | RSNC | QRsnc-4B.1 |  | 4B |  |  |
| 148 |  | SNUE | QSnue-4B.2 |  | 4B |  |  |
| 149 |  | TNUE | QTnue-4B.3 |  | 4B |  |  |
| 150 |  |  | QTnue-4B.4 |  | 4B |  |  |
| 151 |  | PH | QPh-4B.1 |  | 4B |  |  |
| 152 |  |  | QPh-5B.3 |  | 5B |  |  |
| 153 |  |  | QPh-6D |  | 6D |  |  |
| 154 |  | SN | QSn-4A |  | 4A |  |  |
| 155 |  |  | QSn-4B.2 |  | 4B |  |  |
| 156 |  |  | QSn-4B.3 |  | 4B |  |  |
| 157 |  |  | QSn-5A |  | 5A |  |  |
| 158 |  | GN | QGn-1A |  | 1A |  |  |
| 159 |  |  | QGn-4B |  | 4B |  |  |
| 160 |  |  | QGn-5B.2 |  | 5B |  |  |
| 161 |  | SL | QSl-2A.1 |  | 2A |  |  |
| 162 |  |  | QSl-2A.2 |  | 2A |  |  |
| 163 |  |  | QSl-2D |  | 2D |  |  |
| 164 |  | TSS | QTss-5D |  | 5D |  |  |
| 165 |  |  | QTss-7B |  | 7B |  |  |
| 166 |  |  | QTss-7D.2 |  | 7D |  |  |
| 167 |  | BSSS | QBsss-1A.1 |  | 1A |  |  |
| 168 |  |  | QBsss-7A |  | 7A |  |  |
| 169 |  | GWP | QGwp-4B |  | 4B |  |  |
| 170 |  | StWP | QStwp-2B |  | 2B |  |  |
| 171 |  |  | QStwp-4B |  | 4B |  |  |
| 172 |  | AWP | QAwp-1D |  | 1D |  |  |
| 173 |  |  | QAwp-4B.2 |  | 4B |  |  |
| 174 |  | GNC | QGnc-1D |  | 1D |  |  |
| 175 |  |  | QGnc-5A.2 |  | 5A |  |  |
| 176 |  | ANC | QAnc-5A.2 |  | 5A |  |  |
| 177 |  |  | QAnc-5A.3 |  | 5A |  |  |
| 178 |  | GNUE | QGnue-4B |  | 4B |  |  |
| 179 |  |  | QGnue-6A.4 |  | 6A |  |  |
| 180 |  | ANUE | QAnue-4A |  | 4A |  |  |
| 181 |  |  | QAnue-4B.3 |  | 4B |  |  |
| 182 | Maize | ASI - HN | ppr | 181 doubled haploid (DH) | 1 | 62,077 SNP | Sanchez *et al*. 2023 |
| 183 |  |  | vap726 |  | 1 |  |  |
| 184 |  |  | hb64 |  | 1 |  |  |
| 185 |  |  | hsftf2 |  | 2 |  |  |
| 186 |  |  | bhlh20 |  | 2 |  |  |
| 187 |  |  | nup58 |  | 2 |  |  |
| 188 |  | PHT - LN | umc2230 |  | 1 |  |  |
| 189 |  |  | vq6 |  | 1 |  |  |
| 190 |  | PHT - HN | poll1 |  | 3 |  |  |
| 191 |  |  | pds5a |  | 3 |  |  |
| 192 |  |  | snrkII4 |  | 3 |  |  |
| 193 |  |  | psbs1 |  | 3 |  |  |
| 194 |  | YLD - HN | aco5 |  | 2 |  |  |
| 195 |  |  | cdpk3 |  | 2 |  |  |
| 196 |  |  | prh79 |  | 2 |  |  |
| 197 |  |  | glr3.4 |  | 2 |  |  |
| 198 |  |  | mate21 |  | 2 |  |  |
| 199 |  |  | pdi12 |  | 2 |  |  |
